# *Klebsiella* phage KP34gp57 capsular depolymerase structure and function: from a serendipitous finding to the design of active mini-enzymes against *K. pneumoniae*

**DOI:** 10.1128/mbio.01329-23

**Published:** 2023-09-14

**Authors:** Barbara Maciejewska, Flavia Squeglia, Agnieszka Latka, Mario Privitera, Sebastian Olejniczak, Paulina Switala, Alessia Ruggiero, Daniela Marasco, Eliza Kramarska, Zuzanna Drulis-Kawa, Rita Berisio

**Affiliations:** 1 Department of Pathogen Biology and Immunology, University of Wrocław, Wrocław, Poland; 2 Institute of Biostructures and Bioimaging, CNR, Napoli, Italy; 3 Department of Pharmacy, University of Naples Federico II, Napoli, Italy; Columbia University Medical College, New York, New York, USA

**Keywords:** capsule depolymerase, phage-borne enzyme, crystal structure, mini-enzyme, carbohydrate degrading enzymes

## Abstract

Virion-associated depolymerases are large trimeric and multi-domain proteins that constitute the phage arsenal to degrade the polysaccharide layers in their bacterial host. Thus, as recombinant proteins, they are endowed with huge potential in biotechnology and medicine. In this study, we elucidated the structural and functional features of the capsular depolymerase KP34gp57 from the *Klebsiella* phage KP34. Based on the crystal structure and site-directed mutagenesis, we localized the key catalytic residues in an intra-subunit deep groove. Moreover, we engineered several N- and C-terminally truncated versions of KP34gp57 to dissect the role of each domain in the enzyme’s stability and catalytic activity. Serendipitously, our studies revealed C-terminally trimmed KP34gp57 variants that did not trimerize and were sufficiently stable to preserve full catalytic activity as monomers. The elaboration of trimmed monomeric and fully active phage depolymerases is innovative in the field, as no previous example exists apart from bacterial enzymes. Mini phage depolymerases can be optionally combined within chimeric enzymes to extend their activity range, facilitating their use in stand-alone treatments. Moreover, the intra-subunit and inter-subunit locations of the catalytic pocket in phage depolymerases might suggest differences in their evolutionary origin.

**IMPORTANCE:**

In this work, we determined the structure of *Klebsiella* phage KP34p57 capsular depolymerase and dissected the role of individual domains in trimerization and functional activity. The crystal structure serendipitously revealed that the enzyme can exist in a monomeric state once deprived of its C-terminal domain. Based on the crystal structure and site-directed mutagenesis, we localized the key catalytic residues in an intra-subunit deep groove. Consistently, we show that C-terminally trimmed KP34p57 variants are monomeric, stable, and fully active. The elaboration of monomeric, fully active phage depolymerases is innovative in the field, as no previous example exists. Indeed, mini phage depolymerases can be combined in chimeric enzymes to extend their activity ranges, allowing their use against multiple serotypes.

## INTRODUCTION

The World Health Organization (WHO) has identified antimicrobial resistance (AMR) as one of the three greatest threats to mankind. Antibiotic resistance is reaching dangerously high levels worldwide. New AMR mechanisms are emerging and spreading, threatening the ability to treat various infectious diseases as antibiotics become less effective. WHO published the list of antibiotic-resistant “priority pathogens,” distinguishing three types of critical bacteria with the highest priority for investment in new drugs. Priority 1 (CRITICAL) types include carbapenem-resistant *Pseudomonas aeruginosa*, *Acinetobacter baumannii*, and AMR Enterobacteriaceae (1, 2). Among these, *Klebsiella pneumoniae* with emerging extended spectrum β-lactamase-producing and carbapenem-resistant (CRKP) strain is recognized as the third leading pathogen of mortality worldwide (>600,000 deaths, 19.9% antimicrobial resistance-attributed deaths) (3). It is mainly associated with nosocomial infections and has recently been reported as a high risk for coronavirus disease 2019 patients requiring mechanical ventilation and treatment in intensive care units (4). Apart from AMR characteristics, *K. pneumoniae* is equipped with several virulence factors, mainly associated with a cell envelope shield protecting bacteria from harmful environmental factors and the immune system’s response (5). This complex structure exposes two surface glycans: capsular polysaccharides (CPS, the K antigen) and lipopolysaccharides (LPS, the O antigen). CPS is a highly diverse structure, and there are at least 79 structurally defined *Klebsiella* CPS serotypes differing in the composition of sugars and the properties of glycosidic linkages. Moreover, based on *cps* locus sequence analysis, 186 genotypes (KL types) have already been described (6 – 8). There is a strong correlation between CPS overproduction and hypervirulence in *K. pneumoniae*, since poorly capsulated strains or capsule-deficient mutants are more efficiently phagocytized by macrophages and neutrophils (9 – 15). Both CPS and LPS can also be engaged in protecting against serum complement activity (16, 17). Therefore, effective polysaccharide-degrading agents represent a potential non-traditional treatment for combating encapsulated pathogenic bacteria (18).

Polysaccharide depolymerases encoded by *Klebsiella*-specific phages are of great interest in this regard, although they are highly specific toward a particular CPS serotype. Those virion-associated enzymes incorporated into tailspikes are responsible for the recognition, binding, and degradation of *Klebsiella* surface polysaccharides during the phage infection process (18 – 22). Applied externally as recombinant proteins, they deprive bacteria of the capsule, reduce virulence, and sensitize them to antibiotics and the immune system (18, 21, 22). There is profuse literature on the biological activity of depolymerases acting against *K. pneumoniae*, showing effective antivirulence and antibacterial effects both *in vitro* and *in vivo* (21, 23 – 35). On the other hand, only a few studies relate to the architecture and in-depth knowledge at the structural and molecular level; thus, knowledge of the domain organization modes and the mechanisms of CPS cleavage by *Klebsiella* phage-borne depolymerases is still scarce (25, 36, 37). Being virion-associated elements of tailspikes, these enzymes are homotrimers, with each monomer containing parallel right-handed β-helices and consisting of at least three functional domains: the N-terminal baseplate binding domain, the central β-helix domain (with catalytic activity), and a C-terminal domain, which often encodes chaperones for folding and trimerization and/or may be involved in host recognition (35, 38). It is highly likely that, due to the flexibility and hydrophobicity of their N-terminus, recombinantly prepared phage depolymerases can be prone to aggregation in solution (39). The catalytic pocket of phage depolymerases can generally be placed between two β-helices of two neighboring monomers (inter-subunit location) or on the single β-helix domain (intra-subunit location). In both cases, the natural "groove" that fits into a substrate must be provided (40). In our previous study, we presented mechanistic insights on KP32gp38, which consists of four domains [N-terminal, catalytic, carbohydrate-binding module (CBM), and C-terminal lectin domains]. Structural analysis suggested the inter-subunit location of the catalytic pocket; thus, the trimerization of KP32gp38 was essential to maintaining the enzymatic function (25). Such an arrangement of the catalytic center hinders the modifications of a recombinant protein, which could affect trimerization and, consequently, lead to the loss of enzymatic activity. In contrast, the intra-subunit location of the active site allows the preparation of mini-enzymes (e.g., single monomers or enzymatically active domains only), which are desired for biotechnological or medical applications. Smaller-sized enzymes are generally easier to prepare and are characterized by better pharmacokinetics and storage stability.

In this study, we used X-ray crystallography, light scattering, site-directed mutagenesis, mass spectrometry (MS), and additional genetic protein modifications (truncation) to answer several questions related to the structural organization of KP34gp57 depolymerase and the structural determinants of its trimerization and catalysis. This information was precious for the development of elaborately trimmed and fully active mini depolymerases with potentially important uses as anti-infective agents.

## MATERIALS AND METHODS

### Recombinant protein production

The gene encoding the wild-type (WT) depolymerase and all the truncated variants of KP34gp57 were amplified by the polymerase chain reaction (PCR) using Platinum Taq DNA Polymerase, High Fidelity (Invitrogen, Thermo Fisher Scientific, Waltham, MA, USA), phage KP34 DNA as a template, and appropriate primers (Table S1). Subsequently, the PCR products were cloned into the cloning/expression vector pEXP5-CT/TOPO (Invitrogen, Thermo Fisher Scientific) using the manufacturer’s protocol. After the cloning, the constructs were transformed into *Escherichia coli* One Shot TOP10 chemically competent cells (Invitrogen, Thermo Fisher Scientific). Successively, the constructs were isolated using the QIAquick Plasmid Miniprep kit (Qiagen, Hilden, Germany) and sequenced by a commercial company (Genomed, Warsaw, Poland). Validated constructs were then transformed into *E. coli* BL21(DE3)pLysS expression strain (Invitrogen, Thermo Fisher Scientific). Cells were grown overnight (O/N) under shaking (200 rpm) at 37°C in the presence of ampicillin (100 µg/mL) and then inoculated in fresh Luria Bertani (LB) broth containing the antibiotic for the preparative culture. Once the exponential bacterial growth phase was reached (OD_600_ = 0.7–0.8), cell cultures were induced by adding isopropyl-β-D-thiogalactopyranoside (IPTG; Sigma-Aldrich, St. Louis, MO, USA) to a final concentration of 0.2 mM and allowed to incubate at 18°C for 18 h with agitation. Successively, the cells were harvested by centrifugation at 8,000 × *g* for 15 min at 4°C. For the purification step, the pellets were suspended in 30 mL of lysis buffer A (0.3 M NaCl, 50 mM Tris-HCl, and 2% (vol/vol) glycerol, pH 7.8) and lysed by three freeze-thaw cycles and sonication (3 s ON/5 s OFF pulses for 90 s at 30% amplitude). After sonication, the cell lysates containing recombinant WT depolymerase or its modified variants were centrifuged at 16,000 × *g* at 4°C for 30 min, and the supernatants were filtered using a syringe PES filter of 0.2 µm. All variants were purified using the same strategy, which involved the use of affinity chromatography combined with molecular exclusion chromatography. The recombinant proteins tagged by 6× histidine on the C-terminus were purified on a Ni^2+^-derivatized His-Trap column (GE Healthcare, Chicago, IL, USA). After washing with 30 vol of lysis buffer A, the proteins were eluted with lysis buffer A containing 300 mM imidazole. The proteins were further purified by gel filtration on Superdex 200 (GE Healthcare, Chicago, IL, USA) with a buffer composed of 150 mM NaCl, 50 mM Tris-HCl, and 2% (vol/vol) glycerol, pH 7.8. The eluted proteins were checked by SDS-PAGE gel, and protein concentrations were determined by the Qubit Protein Assay Kit (Thermo Fisher Scientific) and simultaneously spectrophotometrically on the Implen NanoPhotometer NP80 (Implen GmbH, Munich, Germany).

### CD spectroscopy

Circular dichroism (CD) spectra were recorded with a Jasco J-810 spectropolarimeter equipped with a Peltier temperature control system (Model PTC-423-S; Jasco, Italy). Molar ellipticity per mean residue, [θ] in deg cm^2^•dmol^−1^, was calculated from the equation: [θ] = [θ]obs•MRW•(10•l•C)−1, where [θ]obs is the ellipticity measured in degrees, MRW is the mean residue molecular mass, C is the protein concentration in g/L, and l is the optical path length of the cell in cm. Far-UV measurements (183–250 nm) were carried out at 20°C using a 0.1 cm optical path length cell and a protein concentration of 0.2 mg/mL. The mean residue molecular mass is calculated from MRW = M / (N − 1), where M is the molecular mass of the protein (in kDa) and N is the number of amino acids. The denaturation processes of the variants were investigated by recording the CD signal at 222 nm.

### Light scattering experiments

Light scattering (LS) experiments were performed for the analysis of protein oligomeric states. A MiniDAWN Treos spectrometer (Wyatt Instrument Technology Corp., Santa Barbara, CA, USA) equipped with a laser operating at 658 nm connected online to a size-exclusion chromatography was used. Purified proteins were analyzed by size-exclusion chromatography connected to a triple-angle light scattering detector equipped with a QELS module (quasi-elastic light scattering) for mass and Rh (Hydrodynamic Radii) measurements. Then, 1.5 mg of each sample was loaded on an S200 10/300 column and equilibrated in 50 mM Tris-HCl, 150 mM NaCl, and 5% (vol/vol) glycerol (pH 8.0). A constant flow rate of 0.5 mL min^−1^ was applied. Data were analyzed using the Astra 5.3.4.14 software (Wyatt Technology, Toulouse, France).

### Limited proteolysis and mass spectrometry

MS-based protein footprinting was carried out through limited proteolysis and consequent liquid chromatography–mass spectrometry (LC-MS) analysis. Tryptic hydrolyses were performed by adding TPCK-treated trypsin (1 µg/µL) to aliquots (100 µL) of full-length KP34gp57 (81 µM) at three different ratios of trypsin/KP34gp57: 1:25, 1:100, and 1:1,000 (wt/wt), incubating samples at 37°C for 2 h. Enzymatic digestion was blocked by adding 80 µL of aqueous 0.1% trifluoroacetic acid, and samples were centrifuged (15,800 × *g*, 15 min). LC-M analysis was carried out on an LTQ XL mass spectrometry system (Thermo Fisher Scientific) equipped with a HESI source operating at a needle voltage of 3.5 kV and a temperature of 275°C. Mass calibration was carried out automatically utilizing selected multiply charged ions using a commercial standard mixture of caffeine, Met-Arg-Phe-Ala peptide, and Ultramark (Thermo Fisher Scientific). Multi-charge spectra were deconvoluted using the BioMass program implemented in the Bioworks 3.1 package provided by the manufacturer.

### Crystallization and data collection

Purified full-length KP34gp57 was concentrated to ~8 mg/mL, and crystallization trials were performed at 20°C by a hanging-drop vapor-diffusion method using commercially available crystallization screen kits (Index and Crystal Screen I and II; Hampton Research, Aliso Viejo, CA, USA). Crystals appeared after a long time. Once the successful condition was identified, it was further refined manually by adjusting the concentrations of the protein and precipitating agent. Crystals suitable for X-ray diffraction were obtained by mixing 1.0 µL of protein solution concentrated at 5.8 mg/mL with an equal volume of reservoir solution containing 0.04 M citric acid, 0.06 M BIS-TRIS propane pH 6.4, and 20% wt/vol polyethylene glycol 3350. Diffraction data were collected at the ESRF (Grenoble, France) at 100 K. Cryoprotection of the crystals was achieved by rapid soaking in a solution consisting of 0.03 M citric acid, 0.045 M BIS-TRIS propane pH 6.4, 15% wt/vol polyethylene glycol 3350, and 25% (vol/vol) glycerol. Diffraction images were processed using HKL2000 (41).

### Crystal structure determination and refinement

The crystal structure of the enzyme was solved by single-wavelength anomalous dispersion (SAD) using the anomalous signal from the Se atoms of the selenomethionine-labeled protein. The program SOLVE (42) was used to localize the selenium sites present in the asymmetric unit and to derive the experimental phases. Phases were improved by density modification using the program RESOLVE (42). Crystallographic refinement was first carried out against 95% of the measured data using the CCP4 program suite (43). The remaining 5% of the observed data, which were randomly selected, was used in Rfree calculations to monitor the progress of refinement. The refinement in Refmac started with data up to 2.5 Å resolution and increased in successive rounds of refinement to the highest resolution (44). The bulk solvent was modeled based on Babinet’s principle, as implemented in the Refmac program. The final round of refinement was carried out with the inclusion of riding H atoms for protein residues. The structure was validated using the program PROCHECK (45).

### *In silico* structural predictions

A reliable three-dimensional model of the C-terminal domain of KP34gp57 was generated by artificial intelligence (AI) using the Colab server (46). This server predicts protein structures starting from their sequences using a slightly simplified version of AlphaFold v2.0 that does not consider existing structural templates. The reliability of the AF predictions was assessed by the Local Distance Difference Test (LDDT) score reported for each structure. Both experimental and predicted structures were inspected by molecular graphics and using available software such as PyMol to identify secondary structure elements and domain boundaries and Dali (http://ekhidna2.biocenter.helsinki.fi) to detect structural similarities with structures deposited in the Protein Data Bank (PDB) (47 – 49).

Proteins containing insertion domains similar to KP34gp57 (YP_003347651.1) and LKA1gp49 (YP_001522890.1) were searched using Dali (http://ekhidna2.biocenter.helsinki.fi) or BlastP in cases of unknown structures (50). In this latter case, modeling was performed using RoseTTAFold (51).

### Site-directed mutagenesis of catalytic residues

Residues involved in the catalysis were predicted based on the structure analysis and type of amino acid (highly evolutionary conserved glutamic acids, aspartic acids, arginine, and tyrosine located in the catalytic domain and/or insertion domain of the KP34gp57). To ascertain the individual roles in catalysis, the selected amino acids of KP34gp57 (R145, D151, D179, D219, Y220, D224, E266, E300, D382, Y384A, and D389) were modified into alanine or isosteric residues (in the absence of expression for alanine scanning) and produced as a single, double, or triple mutant (see Table 2; Table S2). Substitution mutations were introduced using the GeneArt Site-Directed Mutagenesis System and manufacturer protocol (Invitrogen, Thermo Fisher Scientific). Appropriate complementary primers (Table S2), AccuPrime Pfx DNA polymerase, and the pEXP5-CT/TOPO vector (Invitrogen, Thermo Fisher Scientific) containing a WT KP34gp57 gene as a template were used for mutagenic PCRs. After mutagenesis, purified vectors were transformed into *E. coli* One Shot MAX Efficiency DH5α-T1^R^ competent cells (Invitrogen, Thermo Fisher Scientific). Transformed strains were isolated by selective plating, and the amplified plasmids were purified and sequenced by a commercial company (Genomed, Warsaw, Poland) to confirm the designed substitutions. Plasmids with the correct mutations were transformed into *E. coli BL21*(DE3)pLysS (Invitrogen, Thermo Fisher Scientific) for protein expression. All protein mutants were expressed and purified under the same conditions as the wild-type enzyme.

### Activity assays of recombinant proteins on *K. pneumoniae* lawn

The CPS-degrading activity of the purified enzymes was verified by a semi-quantitative assay on a bacterial lawn in a two-fold dilution series similar to the recommendations concerning the determination of the minimum inhibitory concentration (MIC) of drugs. For WTs, catalytic site mutants, and truncated versions, a sample of freshly purified protein at a concentration of 1.5 µM was established using phosphate-buffered saline solution, pH ~7.4 as a diluent. Subsequently, serial two-fold dilutions were prepared to the last concentration of 0.18 nM, and a volume of 10 µL of each concentration was spotted on *K. pneumoniae* 77 (K63 serotype) lawn. In the case of catalytic site mutants, higher concentrations (3, 7.5, 11, 15, 22, and 30 µM) were prepared and tested as well. The minimal halo-forming unit (MHFC) was set as the lowest protein concentration causing a still visible, transparent halo zone on a bacterial lawn after 18 h of incubation at 37°C (examples in Fig. S1) (25) (52). MHFC enzymatic assays were performed in two replicates. To control the K63 serotype specificity of prepared enzymes, we used other *K. pneumoniae* strains with genetically confirmed K63 locus type, including the NCTC 9183 strain from the National Collection of Type Cultures, UK, and four clinical isolates from the collection of the Department of Pathogen Biology and Immunology, University of Wroclaw, Poland.

### Extraction of bacterial CPS from *Klebsiella* K63 serotype

The extraction and purification of CPS from *Klebsiella* K-type 63 was done according to the protocol described previously (53). Twenty milliliters of each overnight *K. pneumoniae* culture in Tryptic Soy Broth (TSB; bioMerieux, Marcy l’Etoile, France) was added to a flask with a broad bottom containing 200 mL of TSB (to provide a large surface for biofilm formation). Biofilm was grown for 5 days at 37°C in static conditions. After biofilm development, to prevent cell lysis, 1,200 µL of formaldehyde was added, and the mixture was incubated at room temperature (RT) with shaking for 1 h. The addition of 80 mL of 1 M NaOH, followed by a 3-hour long incubation with shaking, led to the exopolysaccharide extraction. The supernatant containing exopolysaccharides was separated by centrifugation (16,800 × *g*, 1 h, 4°C) and dialyzed overnight against distilled water (12–14 kDa molecular weight cut-off membrane; SERVA Electrophoresis GmbH, Heidelberg, Germany). The addition of trichloroacetic acid (TCA; SERVA Electrophoresis Gmbh) to the final concentration of 20% and incubation on ice for 0.5 h were performed for protein and nucleic acid precipitation. Subsequently, 1.5 vol of cold 96% ethanol (VWR, Radnor, PA, USA) was added to the supernatant and separated by centrifugation (16,800 × *g*, 1 h, 4°C) in order to precipitate exopolysaccharides from lipids during a 24-hour incubation at −20°C. Exopolysaccharides were centrifuged (16,800 × *g*, 1 h, 4°C), the supernatant was discarded, and the pellet was resuspended in ultrapure water. The CPS solution was dialyzed against Milli-Q water (12–14 kDa molecular weight cut-off membrane’ SERVA Electrophoresis Gmbh) overnight, followed by lyophilization. Lyophilized CPS were resuspended in ultrapure water to the desired concentration.

### Enzymatic assay on extracted *K. pneumoniae* CPS

The enzymatic activity of different variants of *KP34gp57* (300 µg for each) was evaluated in a reaction buffer containing 50 mM Tris-HCl pH 7.8, 150 mM NaCl, and 1.5 mg of CPS as substrate. The reactions were incubated at 37°C for 5 h, and the resulting amounts of reducing sugars were evaluated by the dinitrosalicylic acid (DNS) method (54). DNS reagent was prepared by adding 0.5 g of 3,5-dinitrosalicylic acid, 0.5 g of NaOH, 200 mg of crystallin phenol, 25 mg of sodium sulfite, and 20 g of potassium sodium tartrate in 50 mL of water. Equal volumes of DNS reagent and enzyme-treated products were mixed together and then heated at 100°C for 10 min. After cooling to room temperature, the optical density at 575 nm was measured using a spectrophotometer. Reducing sugar concentrations were determined from a standard curve drawn using absolute glucose ranging from 0.4 to 1.5 mM. All enzymatic assays were performed in triplicate.

### CPS degradation analysis by mass spectrometry

For the mass spectrometry analysis of the reaction products, the previously described reactions were further treated by adding trypsin for 2 h at room temperature and heating to 100°C for 10 min. The denatured depolymerases were removed by centrifugation at 14,200 rpm, and the digested products, which were present in the supernatant, were lyophilized for subsequent chemical structure analysis by mass spectrometry. The absorption of the samples was analyzed at 490 nm and compared with a calibration curve measured with known concentrations of glucose. CPS fragments were analyzed by tandem mass spectrometry (MS/MS). The general MS/MS conditions were as follows: spray voltage = 3.5 kV; vaporizer temperature = 350°C; sheath gas (nitrogen) = 10; auxiliary gas (nitrogen)flow = 2 arbitrary units; ion transfer capillary temperature = 350°C; and positive ion polarity. Fragmentation was induced by a single ion.

## RESULTS

### Serendipitous crystal structure of KP34gp57 monomeric depolymerase

The CPS depolymerase KP34gp57 is a large enzyme with a trimeric organization consisting of 630 amino acid residues per chain and an MW of the trimer of ~200 kDa (Fig. S2). Sequence analysis of KP34p57 does not identify homologous proteins of known structure in the PDB. However, bioinformatic analysis using BetaWrap (55) clearly detects a β-helical structure in the region embedding residues 57–241 (*P*-value 0.0021), which is a typical structural motif of phage tailspike depolymerase catalytic domains.

**FIG 1:**
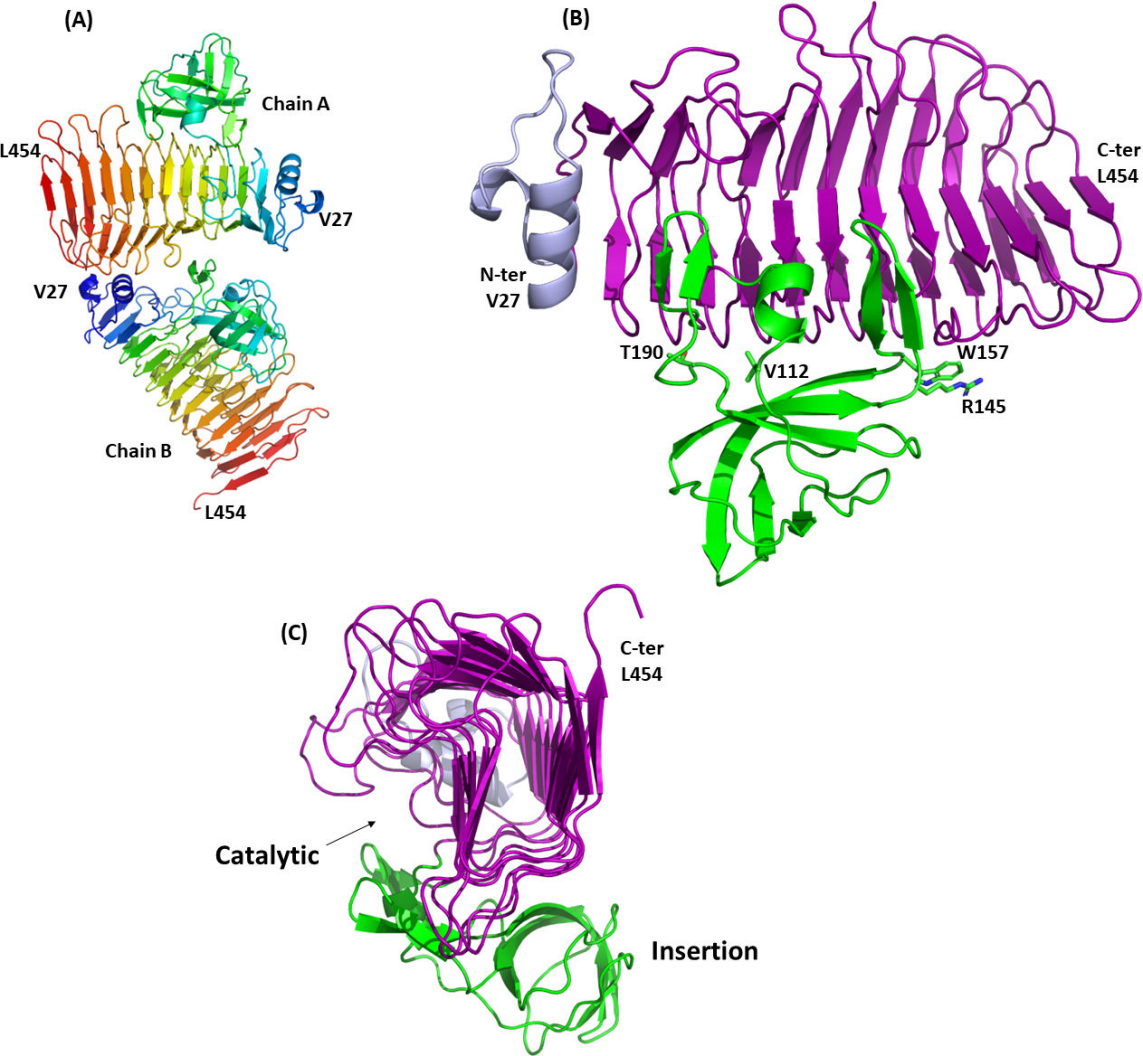
Cartoon representations of the KP34gp57 crystal structure show (**A**) the spacial organization of the two non-crystallographically related molecules in the unit cell. Molecules are represented in rainbow color, from blue (N-terminus) to red (C-terminus); (**B and C**) the domain dissection of chain A in the N-terminal domain (light blue, residues 27–56), the β-helix domain (purple, residues 57–102 and 204–454), and the insertion domain (green, residues 103–203). Panel C highlights the deep groove formed by the β-helix and the insertion domain.

Well-diffracting crystals of KP34gp57 were successfully obtained using polyethylene glycol 3350 solutions as precipitants. A Se-Met derivative was prepared for structure determination using single-wavelength anomalous dispersion (SAD). Surprisingly, we collected diffraction data at 2.0 Å resolution, which produced a unit cell and space group (*a* = 38.3, *b* = 141.0, *c* = 81.9; *β* = 102.6, space group P2_1_) not compatible with the size of the protein (Table 1). Even with one trimer in the asymmetric unit, we measured Matthew’s coefficient Vm outside the allowed range and a negative solvent content. Therefore, it appeared obvious that KP34gp57 had encountered some proteolytic degradation prior to crystallization. Only upon experimental structure determination using SAD did electron density maps clearly reveal two copies of the portion 27–454 in the unit cell, i.e., missing the C-terminal domain (Fig. 1A). The N-terminal region of each copy (residues 27–56) contains two α-helices, followed by the typical β-helix structure characterizing the catalytic domain of tailspikes (residues 57–102 and 204–454; Fig. 1B). In addition, a six-stranded β-barrel domain protrudes from the catalytic β-helix between residues 103–203 (Fig. 1B and C). This crystal structure content was fully unexpected since monomeric tailspikes have hitherto never been observed and their trimeric state is believed to be crucial for enzyme stability.

**TABLE 1:** Data collection and refinement statistics^*b*^

Parameter	Value
Data collection	
Space group	P2_1_
Unit-cell parameters *a, b, c* (Å); *β* (°)	38.3, 141.0, 81.9; 90.000, 102.6, 90.000
Resolution range (Å)	30.0–2.2
Total no. of reflections	203,918
No. of unique reflections	49,807
Completeness (%)	87.1 (91.3)
*R*_merge_ (%)^*a*^	11.7 (41.5)
Average *I*/*σ*(*I*)	14.2 (2.8)
Refinement	
Rwork/Rfree (%)^*a*^	19.0/24.0
No. of residues	428
No. of water molecules	419
R.m.s. deviations:	
Bond lengths (Å)	0.01
Bond angles (°)	1.07

^*a*^ *R*_merge_ = Σ *h*Σ*i* |*I*(*h*,*i*) − <*I*(*h*)>| / Σ *h*Σ*i I*(*h*,*i*), where *I*(*h*,*i*) is the intensity of the *i*th measurement of reflection *h* and <*I*(*h*)> is the mean value of the intensity of reflection *h*.

^*b*^ Values in parentheses are for the highest resolution shell, 2.25–2.20 Å.

**FIG 2:**
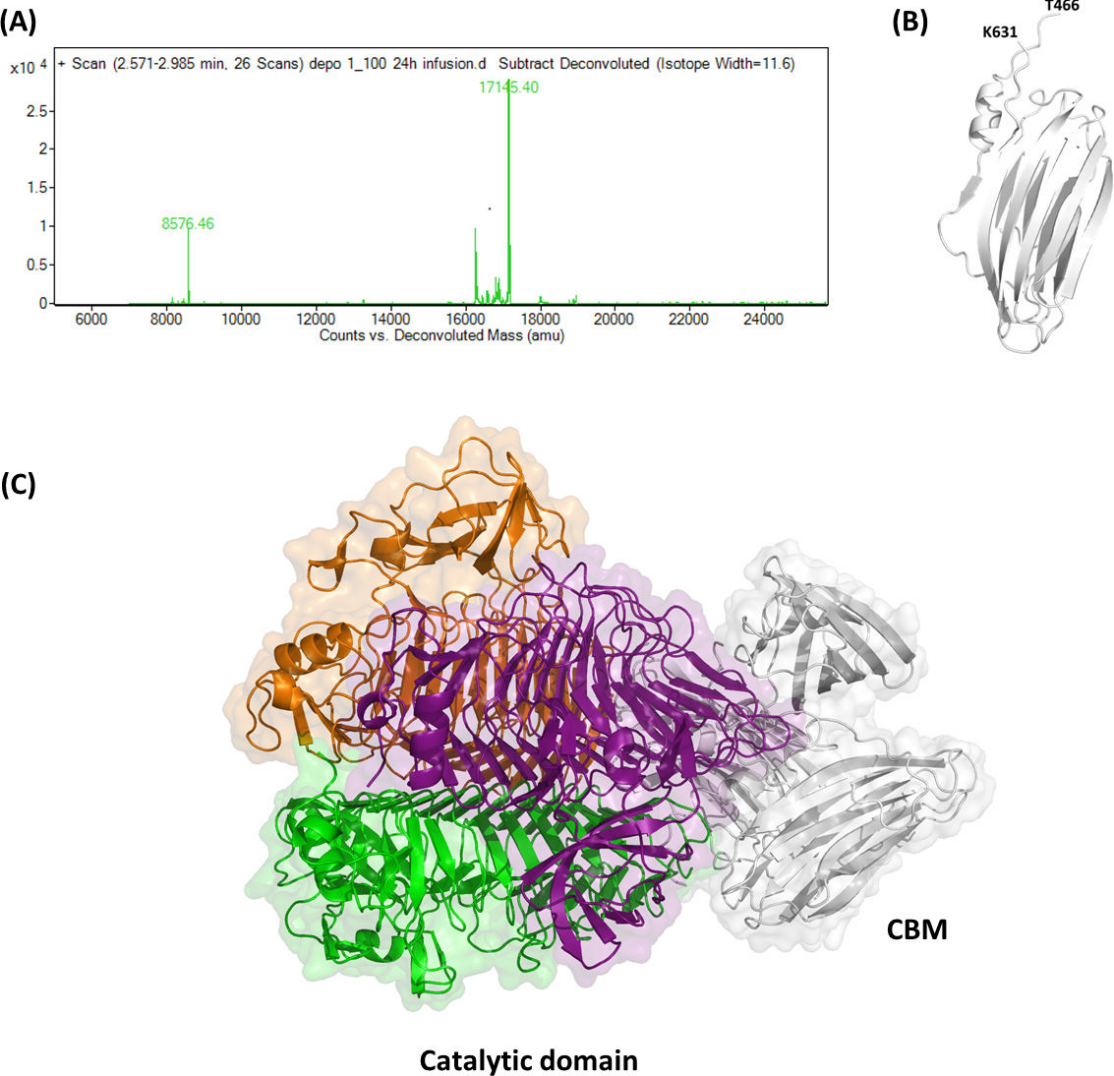
The C-terminal domain (residues 466–631) of KP34gp57. (**A**) Deconvoluted ESI-MS spectrum of the main peak of LC-MS. (**B**) Cartoon representation of the KP34gp57 CBM domain, as determined using AlphaFold, and (**C**) of the whole KP34gp57 protomer, obtained integrating crystallographic data (catalytic domain chains: orange, purple, green) and AlphaFold modeling (CBM domain: gray).

To corroborate our hypothesis of proteolytic digestion, likely due to protease contamination, we used limited trypsin-proteolysis and MS analysis to identify fragments that are more prone to proteolytic degradation. As a result, we observed that all samples deriving from different trypsin to KP34gp57 ratios exhibited the persistence of one chromatographic peak and related electrospray ionization mass spectrometry (ESI-MS) mass spectrum (Fig. 2A), which provided an MW_exp_ = 17,145 amu corresponding to the K^+^ ion-adduct of the C-terminal fragment 466–631 of KP34gp57 (MW_th_ = 17,108 amu). These data confirmed that the C-terminal domain is cleaved off the molecule in all experimental conditions used and shows complete unreactivity to trypsin digestion even at the highest trypsin to KP34gp57 ratio (1:25). Conversely, digested fragments of the N-terminal region, those deriving from 69 to 200 residues, were also evident at the lowest ratio (1:1,000), whereas several stretches from 241 to 280 residues appeared only at high trypsin concentrations (ratio 1:25). These data suggested an increasingly lower flexibility of KP34gp57 from its N- to C-terminal ends.

Given the insignificant sequence identity of the C-terminal domain with proteins of known structure, we used AI and the software AlphaFold 2.0 to predict its structure (46). Results show a reliable model with a high confidence score (pLDDT 90), adopting an asymmetric β-sandwich structure organized in two β-sheets, one with six strands and one with four strands, and resembling CBM domains (Fig. 2B). A model of the entire enzyme obtained combining X-ray crystallography (residues 27–454) and AI (residues 466–631) is reported in Fig. 2C. Despite the low sequence identity, the CBM domain of KP34gp57 is structurally similar to that observed in the crystal structure of KP32gp38 (PDB code 6TKU, DALI Z = 7.8, RMSD = 3.1 Å, seqid 6%) and in the tailspike protein gp63.1 from *E. coli* phage G7C (PDB code 4QNL, DALI Z = 7.8, RMSD = 3.6 Å, seqid 10%) (56). However, their roles, either as CBM or trimerization domain, are hitherto unclear (25, 35, 57). In KP34gp57, the high resistance of this CBM to proteolysis, together with the observation of the monomeric state of KP34gp57 once deprived of the CBM, suggests that the CBM domain plays a crucial role in inducing a compact trimerization of the enzyme while still allowing for the necessary flexibility needed by the catalytic domain for enzymatic catalysis.

### The catalytic site

**FIG 3:**
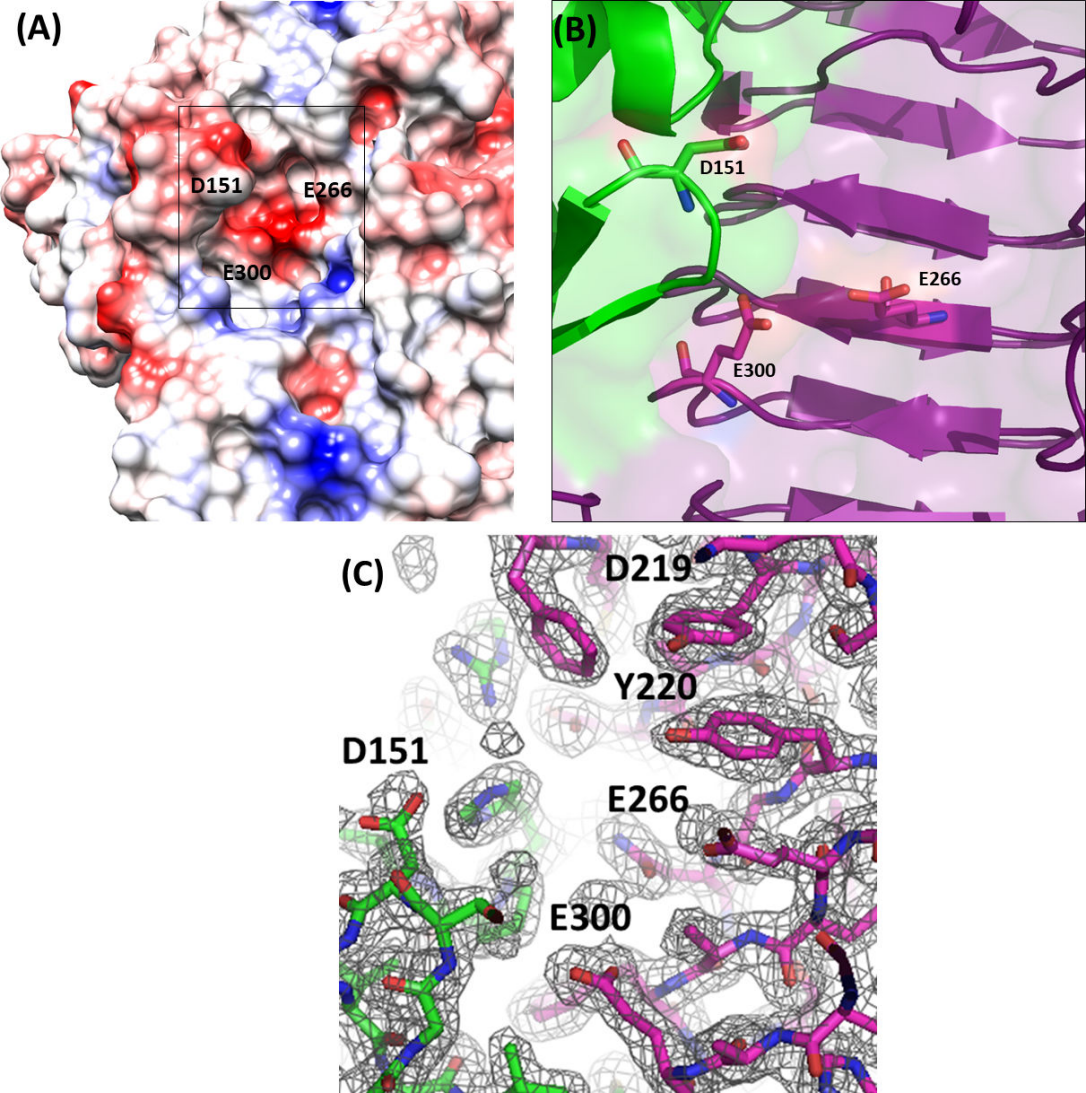
Zoom in on the catalytic pocket of KP34gp57. (**A**) Electrostatic potential surface showing key residues contributing to its charge. (**B**) Cartoon representation of the catalytic cavity, formed by the KP34gp57 β-helix (purple) and the insertion β-barrel domain (green). The predicted key residues for catalysis are shown on a stick. (**C**) (2Fo-Fc) electron density of the catalytic pocket, contoured at 2σ. Only some residues are highlighted for clarity.

An analysis of the crystal structure of KP34gp57 suggests that the catalytic site cleft of the enzyme is located on every single protomer (Fig. 1). Indeed, we identified a large cavity in each promoter (with an area of 280.9 Å^2^ and a volume of 330.5 Å^3^), formed by both the β-helix and the β-barrel insertion domain (Fig. 2A and B; Fig. S3), using the software CASTp (58). Carbohydrate hydrolases always employ the carboxyl groups of two neighboring Asp/Glu amino acid residues, participating in an acid/base mechanism (59 – 61). A clear electron density describes the catalytic pocket, thus allowing for the identification of E266 and E300 (belonging to the β-helix structure) as the most promising catalytic residues and D151 (belonging to the insertion domain) as likely assisting CPS binding (Fig. 3).

To experimentally assess the role of these residues in catalysis, site-directed mutagenesis was adopted to test all mutants for their activity. In addition to the most promising amino acids identified by structural analysis, other highly conserved amino acids with catalytic potential (R145, D179, D219, Y220, D224, D382, Y384, D389) were subjected to mutagenesis. Alanine scanning is the most widely used approach to detect important residues in proteins (62); therefore, the selected amino acids were changed to alanine as the first choice in mutagenesis. However, due to a significant decrease in the yield of production of alanine-mutated proteins, selected E and D residues were changed to their isosteric non-charged residues (Q and N, respectively).

**FIG 4:**
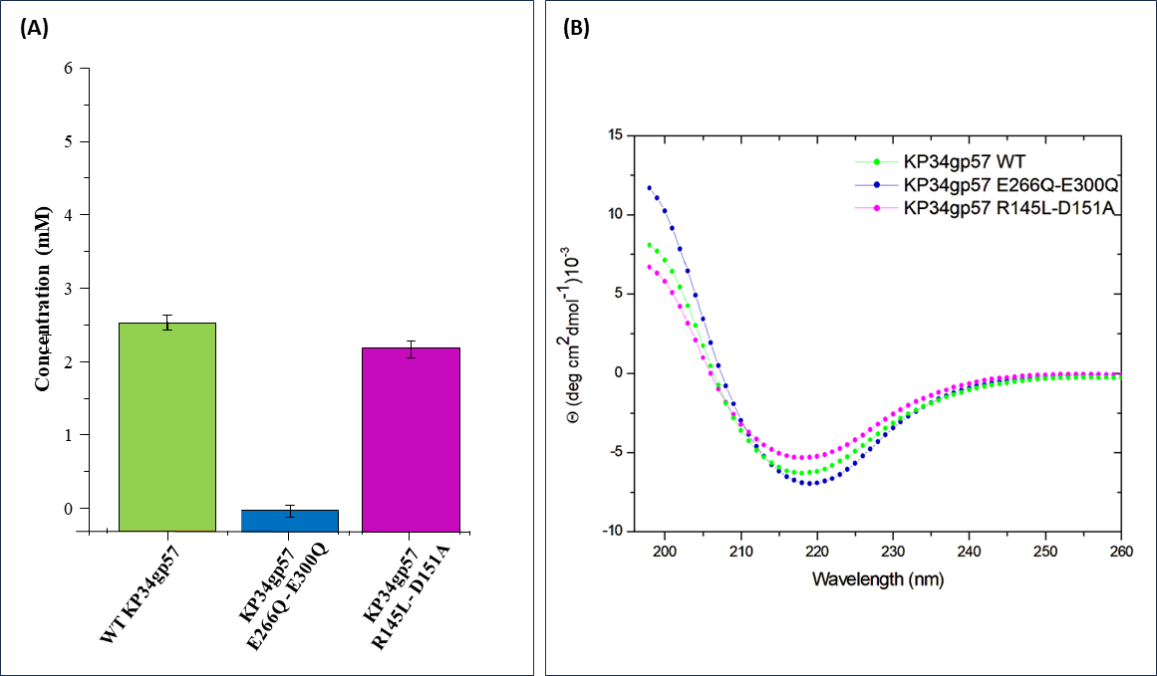
Impact of mutation on catalytic efficacy and structure of KP34gp57. (**A**) Quantitative estimation of catalytic efficacy of mutants using the DNS assay. (**B**) CD spectra of selected mutants compared to those of the wild-type enzyme.

KP34gp57 produces turbid halos on the bacterial lawn, growing upon prolonged incubation due to CPS depolymerization and disaggregation of the cell clumps. The MHFC value was used as a preliminary measure of the depolymerizing activity of protein mutants (Table 2). As a result, the strongest decline in activity was observed for E266A and E300Q single mutants and their double mutants (Table 2). A lower, albeit significant, depression in catalytic activity decrease was also confirmed for mutations of D219, Y220, D224, Y384, and D389 located in the catalytic domain and R145, D151, and D179A in the β-barrel insertion domain (Table 2). Due to the qualitative nature of the MHFC assay, we extracted and purified CPS from *Klebsiella* K-type 63 serotype using the previously reported protocol (53) and quantitatively evaluated the enzymatic activity of selected mutants using the colorimetric DNS method. After incubation of CPS with the enzyme variants, the absorption of all samples was analyzed at 490 nm and compared with a calibration curve measured with known concentrations of glucose. This assay confirmed the crucial role of E266 and E300 in catalysis since E266Q/E300Q is fully inactive (Fig. 4A). Differently, DNS analysis shows that the mutant R145L/D151A is endowed with a slightly, albeit significantly, lower activity than the wild-type enzyme. This suggests that the two residues D151 and R145, belonging to the insertion domain, marginally contribute to catalysis (Fig. 4; Fig. S4). CD spectra of these mutants were recorded to check that the mutations do not affect the structural integrity of the mutated proteins. As shown in Fig. 4B, CD spectra of E266Q/E300Q and R145L/D151A are fully superposable to those of the wild-type enzyme.

**TABLE 2:** Activity of KP34gp57 catalytic cleft mutants, determined by MHFC assay

Enzyme	Expression yield	MHFC (nM)	Activity indicator^*a*^
WT KP34p57	High	23.4	+++
R145L	Medium	46.8	++
R145L - D151A	Medium	375.0	+
D151A	High	375.0	+
D179A	Medium	93.7	+
D151A - D179A	Medium	375.0	+
D219N	Low	750.0	+
Y220A	High	750.0	+
D224A	Medium	187.5	+
E266A	Low	11,000.0	−
E266Q	High	7,500.0	−
E300Q	Medium	1,500.0	+
E266Q–E300Q	Medium	11,000.0	−
D382A	Medium	187.5	+
D382N	High	46.8	++
Y384A	High	93.7	+
D389A	High	187.5	+

^*a*^ Activity indicators were assigned based on the percentage increase in MHFC of the specific variant with respect to WT (MHFC_WT_ / MHFC_variant_ × 100). Specifically: +++ (100%), ++ (>50%), + (1 < MR < 50%), − (MR <1%).

### KP34gp57 degrades CPS into trimeric oligosaccharides

**FIG 5:**
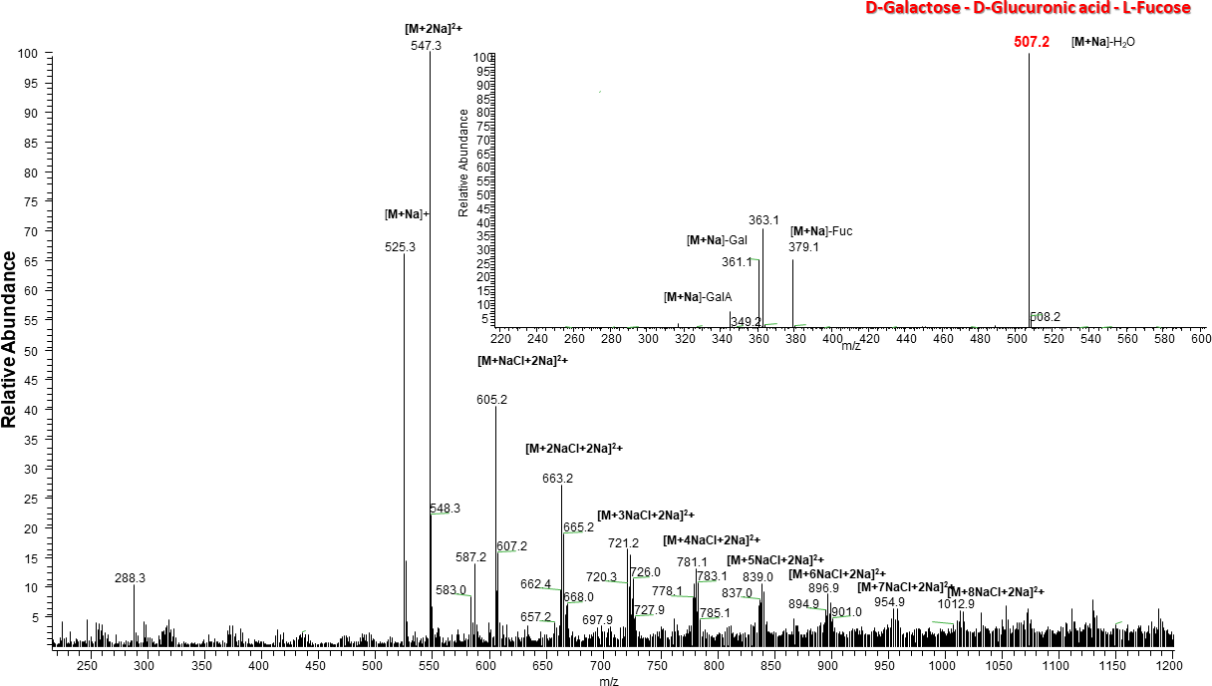
ESI-MS spectrum of an oligosaccharide sample obtained upon extraction and purification of the CPS exopolysaccharides of *Klebsiella pneumoniae*. Inset MS-MS fragmentation of the peak 1,009 amu corresponding to the [(Gal-GalA-Fuc)_2_ + Na]^+^ adduct.

We used ESI-MS to identify the product of CPS digestion by KP34gp57. To achieve this aim, lyophilized CPS exopolysaccharides from *Klebsiella pneumoniae* K-type 63 serotype were resuspended in ultrapure water to the desired concentration for the assay (see Materials and Methods). After incubation of CPS with KP34gp57 at 37°C for 5 h and subsequent removal of the protein, the digested products were lyophilized for subsequent chemical structure analyses by mass spectrometry. The MS spectrum, reported in Fig. 5, presents two main peaks: one at 501 amu, corresponding to the trisaccharide containing galactose, galactutonic acid, and fucose (Gal-GalA-Fuc), and the other at 985 amu, which corresponds to the hexasaccharide (Gal-GalA-Fuc)_2_. Both presented ions deriving from mono-sodiate and NaCl adducts in the presence of NaCl 150 mM in the digestion buffer. MS-MS fragmentation of the main peak (inset of Fig. 5) confirmed the assignment of the hexasaccharide.

### The β-barrel insertion domain is a fingerprint of depolymerases with intra-chain catalytic clefts

A DALI structural analysis (47) carried out using the structure of the KP34gp57 catalytic domain highlights structural similarities with a set of proteins, among several tailspikes. Interestingly, most structurally similar are a set of hydrolases of bacterial origin and monomeric, including *Azotobacter vinelandii* mannuronan C-5 epimerase (e.g., PDB code 5LW3, DALI Z = 28.1, RMSD = 2.5 Å, seqid 12%) and GH87 alpha-1,3-glucanase from *Streptomyces thermodiastaticus* (PDB code 7C7D, DALI Z = 28.1, RMSD = 2.5 Å, seqid 14%). Among tailspikes, most similar are the tailspike of the *Shigella flexneri* Phage Sf6 (2VBK, DALI Z = 27.9, seqid 12%) and gp49 from *Pseudomonas* phage LKA1 (4RU4, DALI Z = 25.1, seqid 15%).

Importantly, several tailspike depolymerases have insertion β-barrel domains similar to those observed in the KP34gp57 structure. These include gp49 from *Pseudomonas* phage LKA1 (PDB code 4RU4; Fig. S5A and B) (DALI Z computed on the insertion domain = 7.0, RMSD = 4.2 Å, seqid 23%), gp61 of *Pseudomonas* phage phi297 (podovirus, PDB code 4RU5, DALI Z = 6.5, RMSD = 3.7 Å, seqid 21%), Gp54 of *Acinetobacter baumannii* phage AP22 (myovirus, PDB code 4Y9V, DALI Z = 7.7, RMSD = 4.2 Å, seqid 19%), and other phage receptor binding proteins (RBPs), e.g., from *Salmonella* phage Det7 (myovirus, PDB code 2V5I) and *Salmonella* phage 9NA (siphovirus, PDB code 3RIQ). Interestingly, in all cases, the catalytic domain is intra-chain, and as observed for KP34gp57, it is completed by the β-barrel insertion domain. This insertion domain is instead not present in tailspikes with inter-chain catalytic pockets, as in the case of the depolymerase KP32gp38 (25), acting against the K21 CPS serotype of *K. pneumoniae* (podovirus, PDB code 6TKU, residues 29–55, DALI Z = 11.1, RMSD = 2.9 Å, seqid 9%) (Fig. S5C and D) and other depolymerases (63).

### Design of short KP34gp57 variants with enhanced properties

Phage depolymerases are generally large trimeric proteins (>150 kDa) characterized by stability in solution, albeit low resistance to storage, a clear obstacle for biotechnological applications. Consistently, we observe that the MHFC of WT KP34gp57 decreases with storage time, both at RT and 4°C, as well as with every freeze/thaw cycle (Table S4). The loss of activity is mainly due to protein aggregation since a gain in activity is obtained upon gel-filtration purification. Therefore, a panel of truncated KP34gp57 versions was designed and prepared to improve protein solubility and reach high yields.

The crystal structure of KP34gp57 revealed no electron density for the first N-terminal 26 residues (Fig. 1), with the N-terminal region being a flexible hydrophobic element anchoring the tailspike (RBP) to the phage particle. Based on this observation, the removal of the N-terminal part should enhance the solubility of prepared proteins. Therefore, 13 different N-terminally truncated versions of KP34gp57 were prepared and analyzed (Table 3; Fig. S6). In some cases (N-G20, N-G35, N-T40, N-V45, N-A50), the trimming resulted in a large drop in the expression efficiency of the protein in its soluble form. However, we obtained six versions (starting at K5, D8, A11, V14, A17, and Q23), which were expressed in amounts comparable to WT KP34gp57, with N-A17 and N-Q23 variants exhibiting increased activity compared to WT KP34gp57 when tested directly after purification (Table 3). The further trimming of the N-terminal helical region (L29-A50) did not improve either the expression yield or the activity of protein batches because it removed a structured or β-helix region. Moreover, WT KP34gp57 and truncated variants N-A17 and N-Q23 diluted to 3 µM in phosphate-buffered saline solution, pH ~7.4, were tested in terms of storage properties. Truncated variants showed a lower level of aggregation than WT KP34gp57 at all tested temperatures (−20°C, 4°C, RT, and 37°C) and during different incubation periods (1, 7, and 30 days) (Table S4). Consistently, structural studies in solution proved that the truncated construct N-A17 presents the same secondary structure and melting temperature as the WT enzyme (Fig. S7A and B), but it is less aggregation-prone (Fig. S7C and D), thus confirming that removal of the short part of the N-terminus has a positive effect on activity/solubility during storage (Table S4).

**TABLE 3:** Activity of truncated KP34gp57 variants, determined by MHFC assay

Enzyme	Length	Truncated end	Number of residues	MW of recombinant protein (kDa)	Expression yield	MHFC (nM)	Activity indicator^*a*^
*WT*	M1- G630		630	67,643.93	High	23.4	+++
*N-terminal truncations*							
*N-K5*	K5-G630	N	627	67,358.59	High	23.4	+++
*N-D8*	D8-G630	N	624	67,018.12	High	23.4	+++
*N-A11*	A11-G630	N	621	66,774.90	High	5.9	**++++**
*N-V14*	V14-G630	N	618	66,389.48	High	5.9	**++++**
*N-A17*	A17-G630	N	615	66,062.10	High	1.5	**++++**
*N-Q23*	Q23-G630	N	609	65,565.53	High	1.5	**++++**
*N-L26*	L26-G630	N	606	65,235.24	High	46.8	++
*N-L29*	L29-G630	N	603	64,874.77	Medium	23.4	+++
*N-R32*	R32-G630	N	600	64,578.41	Medium	46.8	++
*N-G35*	G35-G630	N	597	64,160.94	Low	23.4	+++
*N-T40*	T40-G630	N	592	63,793.54	Low	46.8	++
*N-V45*	V45-G630	N	587	63,334.08	Low	46.8	++
*N-A50*	A50-G630	N	582	62,850.52	Low	46.8	++
*C-terminal truncations*							
*C-G581*	M1-G581	C	581	62,575.28	High	23.4	+++
*C-T522*	M1-T522	C	522	56,620.70	Medium	46.8	++
*C-L469*	M1-L469	C	469	51,066.44	Low	187.5	+
*N- and C-terminal truncations*							
*NC-A17-T522*	A17-T522	N and C	506	55,094.98	Low	23.4	**+++**
*NC-A17-G581*	A17-G581	N and C	566	60,993.45	High	11.7	**++++**

^*a*^ Activity indicators were assigned based on the percentage increase in MHFC of the specific variant with respect to WT (MHFC_WT_ / MHFC_variant_× 100). Specifically: +++ (100%), ++ (>50%), + (1 < MR < 50%), +++ (>100%).

### Structure-based development of a stable and active monomeric mini-enzyme

**FIG 6:**
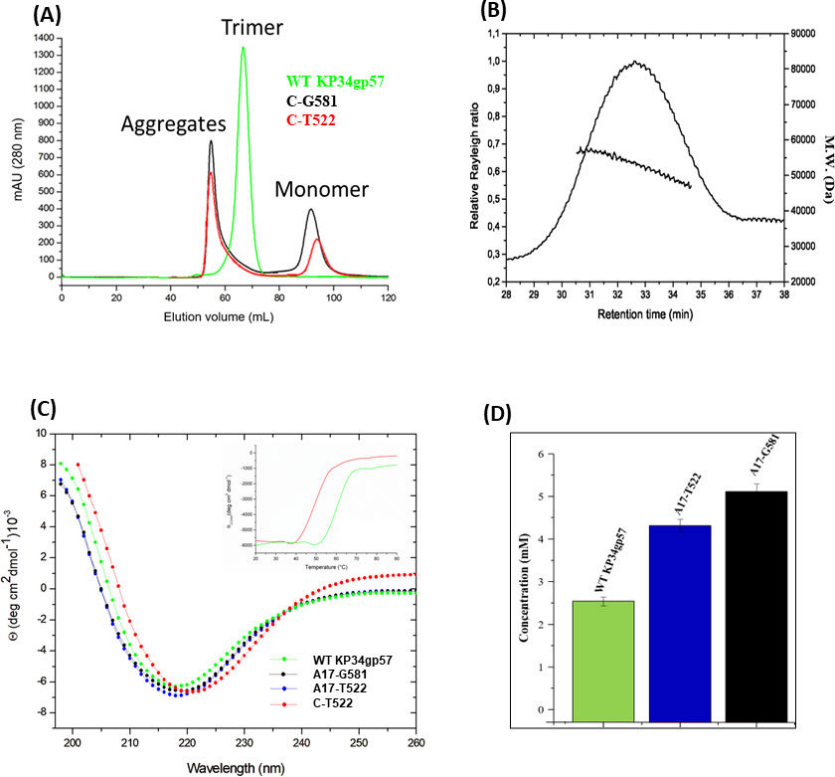
Oligomerization and stability of KP34gp57 variants. (**A**) HPLC profile of WT KP34gp57 (green), C-T522 (red), and C-G581 (black) truncated variants. (**B**) Analytical SEC-LS. Relative Rayleigh ratios (left scale) and derived molecular weight (right scale) versus elution time of C-T522. (**C**) Far-UV CD spectra measured at 0.2 mg mL^−1^ in 20 mM sodium phosphate buffer (pH 7.4); color codes are reported. The inset shows the thermal denaturation curves monitored at 222 nm. (**D**) Quantitative estimation of the catalytic efficacy of truncated variants A17-T522 and A17-G581, compared to WT KPgp57, using the colorimetric DNS assay.

The crystal structure of the KP34gp57 catalytic domain shows that the catalytic sites of the enzyme are located on each promoter. This finding suggested to us that KP34gp57 may also be active as a monomer and pointed to a promising strategy for the development of mini-enzymes. Therefore, we used crystallographic information to design recombinant short monomeric and active enzymes. The crystal structure showed clear electron density, suggesting well-structured residues until Leu-454. Therefore, we designed slightly longer forms, ending with a C-terminal His-tag region for better purification. The first variant (ending at Leu-469; Table 3) showed a lower expression level than the WT enzyme and was strongly aggregation-prone. Likely due to aggregation phenomena, it tended not to bind properly to the Ni^2+^-derivatized His-Trap column, suggesting that the His-tag region was not sufficiently exposed for proper affinity purification. Therefore, we decided to elongate the sequence at the C-terminal region and produced two C-terminally truncated versions, ending at either Thr-522 (C-T522) or Gly-581 (C-G581) (Table 3). As shown in Fig. 6A, both C-terminally truncated forms elute as monomers in HPLC chromatography profiles. Consistently, light scattering and CD spectroscopy analyses showed a monomeric and well-folded state in solution, with a predominant content of β-structure (Fig. 6B and C). In addition, thermal denaturation studies confirmed their high stability with a melting temperature Tm of 50°C, compared to 60°C for WT KP34gp57 (Fig. 6D). A preliminary analysis of catalytic activity using MHFC assays shows that both C-522 and C-581 are active, with a significant activity increase upon further truncation at the N-terminus, as observed for the entire enzyme (Table 3). The resulting NC-A17-G581 and NC-A17-T522 variants were checked for their structural integrity using CD spectroscopy (Fig. 6C) and assayed using DNS for a quantitative evaluation of their catalytic efficacy (Fig. 6D). As a result, we observed that both variants presented a well-folded state (Fig. 6C) and a higher depolymerase activity than the entire enzyme, with the best performance for the NC-A17-G581 variant (Fig. 6D). Indeed, this variant, which was produced in high yields, presents a twofold higher activity than the entire KP34gp57 protein, albeit being characterized by a molecular mass of less than one-third that of KP34gp57 (Table 3).

## DISCUSSION

CPS depolymerases have promising applications as antivirulence agents *in vivo* (64 – 66). However, limitations still exist due to their high specificity toward limited serotypes and their large molecular mass, which often causes poor tissue penetration. Deep knowledge of their structures and mechanisms of action is needed to engineer recombinant proteins with a smaller size and a wider spectrum of action, for instance, by designing chimeric multi-domain enzymes.

In this work, we focused on the structure and function of the CPS depolymerase KP34gp57 derived from *Klebsiella* phage KP34, which efficiently causes a visible halo zone on bacterial lawns of the K63 serotype of *K. pneumoniae* (67). After the extraction of CPS from the K63 serotype, we investigated the hydrolysis reaction by KP34gp57 using mass spectrometry. Our data prove that the visible halo zone produced by KP34gp57 on the bacterial lawn is to be attributed to CPS degradation, and that KP34gp57 degrades the CPS polymer of the K63 serotype down to the trisaccharide (_D_Galactose-_D_Glucuronic acid-_L_Fucose). Interestingly, the serotype K63 is identical to K42 of *E. coli*, a finding that allows the cross-reactive use of KP34gp57 against the two bacteria (68). As a serendipitous finding, the crystal structure of KP34gp57 contained only a portion of the molecule, including the N-terminal and catalytic domains, in a monomeric state due to proteolytic cleavage of the C-terminal domain, which we identified as a CBM domain. The crystal structure of monomeric KP34p57 showed that the catalytic β-helix domain of KP34p57 is interrupted by a six-stranded β-barrel insertion domain, embedded between residues 103 and 203, that contributes to the formation of a large intra-chain catalytic pocket (Fig. 1), with high structural similarity to several proteins of phage and bacterial origin present in the PDB (PDB codes 4RU4, 4RU5, 4Y9V, 7JWF). Interestingly, in all similar proteins, the catalytic domain is intra-subunit and is completed by the β-barrel insertion domain. This finding suggests that the β-barrel insertion domain plays a role in building up a flexible and deep cavity that is able to adapt to a ramified polymer like CPS, a property that is hardly conferred by flat and rigid β-helix structures. Also, combining limited proteolysis with mass spectrometry, we showed that the CBM domain is highly proteolytically stable, suggesting a critical role for this domain in the trimerization of KP34gp57, consistent with the observed monomeric state of the protein in the crystal state (which is deprived of the CBM domain). The strong element of novelty of this result, since phage depolymerases are hitherto known solely as trimeric structures, suggested protein engineering to design smaller and more active enzymes. Based on crystallographic information, we engineered trimmed variants of KP34gp57 (including N-A17 and N-Q23) with high yields and performed better than the wild-type enzyme as far as their storage properties and stability in solution are concerned. Mini versions of KP34gp57 without the CBM domain were prepared and proved to be monomeric and stable forms of KP34gp57 (C-G581, C-T522, and NC-A17-G581) with full activity in *K. pneumoniae* CPS degradation. Also, as observed for the entire trimeric enzyme, its shortened variant lacking the N-terminal residue (NC-A17-G581) was more active than the WT protein when tested directly after gel filtration purification.

A long-lasting dispute exists on the evolutionary hypotheses of phage spikes able to degrade bacterial polysaccharides (CPS/LPS/exopolysaccharide [EPS] depolymerases) (40, 69). A pioneering work showed that the structures of the tailspike of the HK620 phage infecting *E. coli* H, *Shigella* phage Sf6, and *Salmonella* phage P22, which recognize and cleave their respective O antigen LPS, present different binding site locations. The substrate-binding site is intra-subunit in P22 and HK620 tailspikes but inter-subunit in Sf6. This evidence led to the proposal that tailspikes with different specificities may have one ancestor protein, possibly embedding both intra- and inter-subunit binding sites to broaden the host range of their bacteriophage, rather than being formed by the shuffling of unrelated domains in horizontal gene transfer (HGT) events (69). Contextually, Leiman and Molineux (40) proposed two ways of tailspike evolution to adjust new substrates: (i) acquisition of already developed enzymatic domains by HGT or (ii) modification of the catalytic pocket residues. An evolutionary intra- to inter-subunit location switch would require an intermediate form that binds in both locations (40).

Our data added further insight to this dispute based on several considerations. First, they indicate that we are not yet able to predict tailspike enzymatic specificity based on sequence alignment or structural organization. The (CAZy) database (www.cazy.org) currently provides six families of carbohydrate-active enzymes: glycoside hydrolases (GHs), glycosyltransferases (GTs), polysaccharide lyases (PLs), carbohydrate esterases (CEs), auxiliary activities (AAs including lytic polysaccharide monooxygenases, LPMO), and CBMs (73). Among those, the majority of tailspike enzymes found in the PDB database are classified as GHs, including O-antigen endoglycosidases, CPS endosialidases, or other hydrolases. The second abundant tailspike group encompasses PLs, mainly pectate or alginate lyases specific to LPS, EPS, CPS, or biofilm matrix. Interestingly, all pectate lyase structures available in the PDB database show a high topology similarity to tailspikes, regardless of the enzyme origin (viral, bacterial, fungal).

Although some tailspikes degrading the same carbohydrates share high structural homology in at least their catalytic domains, thus suggesting an HGT event between taxonomically distant phages (70, 71), a high structural similarity does not necessarily translate into similar enzymatic specificity. In this study, we found that the catalytic domain of KP34gp57 degrading *Klebsiella* K63 antigen is structurally similar to that of LKA1gp49 despite their different specificities, as LKA1gp49 specifically hydrolyzes *Pseudomonas* O5 antigen. Consistently, proteins like KP34gp57 or LKA1gp49 containing a β-barrel insertion domain exist in a wide range of enzymes distributed among viruses and bacteria, not overlapping in terms of origin with KP34gp57-like proteins (Table S3). Another important element is our observation, acquired here for the first time, that the catalytic domain of intra-subunit depolymerases is sufficient to form an active enzyme. This finding suggests that enzyme trimerization is a tool to provide tailspikes with a dual function, both as enzymes and as structural viral particles. Consistently, we screened the database for the distribution of the monomer-forming homologs, and we found proteins similar to KP34gp57 in viruses (*Klebsiella* and *Serratia* phages), enterobacteria (*Klebsiella* spp. and *E. coli*), α-proteobacteria (*Bradyrhizobium* sp., *Novosphingobium* sp*.,* and *Caulobacter* sp.), and β-proteobacteria (*Cupriavidus* sp.) (Fig. S8). In contrast, homologs to the trimeric enzyme KP32gp38, holding an inter-chain catalytic site, were only distributed in *Klebsiella* phages and enterobacteria (*Klebsiella* spp. and *E. coli*), suggesting prophages origin (Fig. S9).

**FIG 7:**
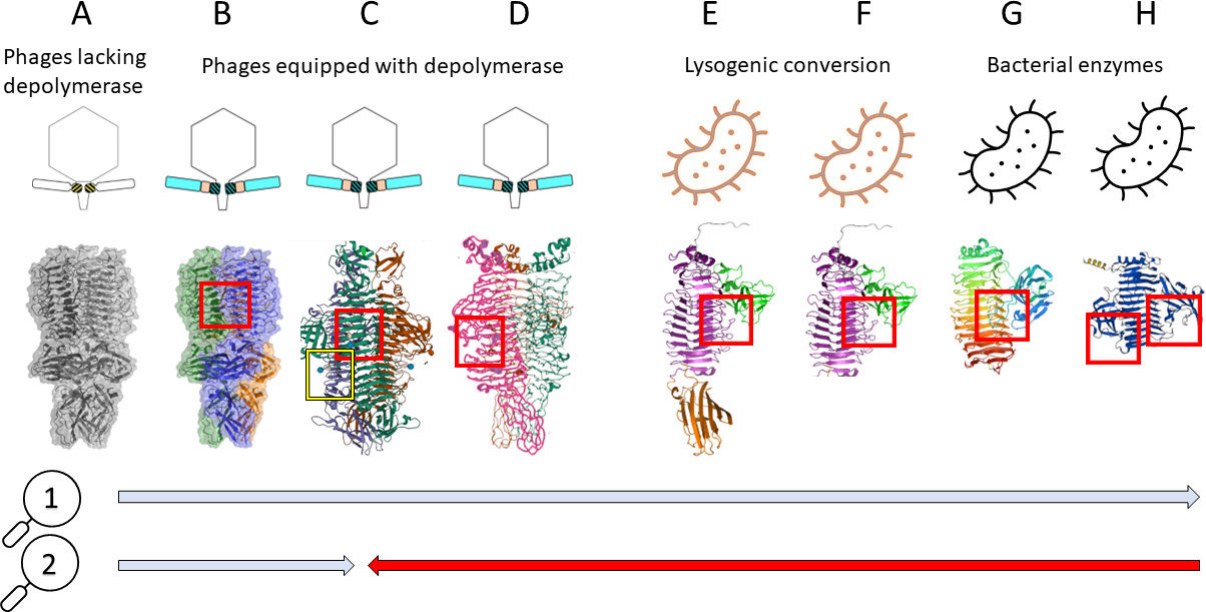
Exopolysaccharide depolymerases evolution hypotheses: Hypothesis 1—from phages to bacteria; Hypothesis 2—from phages to phages (inter-subunit location) and from bacteria to phages (intra-subunit location with an insertion domain forming the groove). (**A**) Phage trimeric tailspike lacking enzymatic activity; (**B**) phage trimeric tailspike gaining an enzymatic feature by forming the catalytic groove between monomers (inter-subunit catalytic pocket; red square; e.g., PDB code 6TKU); (**C**) phage trimeric tailspike bearing two distinct carbohydrate-binding sites encompassing the intra-subunit catalytic pocket (red square) and the inter-subunit non-catalytic carbohydrate-binding site (yellow square) formed by the insertion domain (e.g., PDB code 7W1E)—the intermediate version of (**B**) and (**D**); (**D**) phage tailspike with the intra-subunit catalytic pocket formed by the insertion domain (red square; e.g., PDB codes 8BKE and 4RU4); (**E**) prophage depolymerase equal to the full-domain version of tailspike with the intra-subunit catalytic pocket formed by the insertion domain (red square); (**F**) truncated prophage depolymerase as the monomeric protein with the intra-subunit catalytic pocket formed by the insertion domain (red square); (**G**) bacterial depolymerase as the monomeric protein with the intra-subunit catalytic pocket formed by the insertion domain location equipped with one insertion domain (red square; PDB codes 6K0V and 7C7D); (**H**) *Bacteroides fragilis* YCH46 galactosidase B as the monomeric protein with two distinct intra-subunit catalytic pockets formed by the insertion domains (red squares) (https://www.alphafold.ebi.ac.uk/entry/AF-Q64XV2).

Based on these findings, hypotheses of the possible evolution of enzymes able to degrade bacterial exopolysaccharides (CPS/LPS/EPS) can be drawn (Fig. 7). Possibly, depolymerases are phage-originating proteins that developed the enzymatic activity in trimeric spikes through the modification of key residues that provide a specific inter-subunit substrate groove (Fig. 7A and B). Following this, tailspikes with inter-subunit grooves may have evolved into intra-subunit ones, such as KP34gp57 (D type in Fig. 7), through the HGT acquisition of the β-barrel insertion domain and mutations of newly formed intra-subunit grooves. The *Klebsiella* phage K1 trimeric lyase (Fig. 7C) bearing two distinct carbohydrate-binding sites (the intra-subunit catalytic pocket and the inter-subunit non-catalytic carbohydrate-binding site, PDB code 7W1E) (37) could be an example of the evolutionary connection between tailspikes with inter- and intra-subunit catalytic pockets. Finally, phage depolymerases are acquired by bacteria through lysogenic conversion and may exist in intact prophages as full-version proteins (Fig. 7E) or as catalytic domains only in domesticated prophages (Fig. 7F). The ability of a KP34gp57-like enzyme to be stable and active as a monomer opens the door to an evolutionary accommodation of depolymerases by bacteria through its further adaptation to mini monomeric versions or even encompassing the next insertion domain with the catalytic activity (Fig. 7G and H). A second hypothesis (Fig. 7, Hypothesis 2) is based on the observation of structurally similar and monomeric bacterial enzymes enabling the degradation of CPS, LPS, and biofilm matrix (72). Contrary to the first hypothesis, these enzymes might have been uptaken by phages over the lysogenization process and further modified to acquire the trimeric state mandatory for functional viral tailspikes (Fig. 7).

In conclusion, our results provide multiple insights into the mechanistic action of depolymerase on *K. pneumoniae* bacteria, as they (i) identify molecular determinants of depolymerase stability and resistance to storage, (ii) provide structural and functional clues on the catalytic function of the KP34gp57 enzyme and identify key actors of catalysis, and (iii) prove that depolymerases holding intra-subunit catalytic sites can be stable and active in shorter and monomeric versions. Altogether, our findings deliver tools for protein engineering aimed at overcoming limitations hitherto existing in the use of CPS depolymerases as therapeutics. Finally, they open the door to novel hypotheses on the evolutionary scenario of enzymes able to degrade bacterial exopolysaccharides (CPS/LPS/EPS).

## Data Availability

The atomic coordinates and structure factors of the crystal structure have been deposited in the Protein Data Bank under accession code 8BKE. Other data and materials are available from the corresponding authors on reasonable request.

## SUPPLEMENTAL MATERIAL

Supplemental Tables and Figures mbio.01329-23-s0001.docx Tables S1-S4 and Fig. S1-S9.

ASM does not own the copyrights to Supplemental Material that may be linked to, or accessed through, an article. The authors have granted ASM a non-exclusive, world-wide license to publish the Supplemental Material files. Please contact the corresponding author directly for reuse.
