# Supplementary material for: Klebsiella phage KP34gp57 capsular depolymerase structure and function: from a serendipitous finding to the design of active mini-enzymes against K. pneumoniae
Source: mBio. 2023 Sep 14;14(5):e01329-23. doi: 10.1128/mbio.01329-23 (PMC10653864; doi:10.1128/mbio.01329-23)
Supplement: Supplemental Tables and Figures — Tables S1-S4 and Fig. S1-S9. [file mbio.01329-23-s0001.docx]

**Klebsiella phage KP34gp57 capsular depolymerase structure and function, from a serendipitous finding to the design of active mini-enzymes against *K. pneumoniae***

**Supplementary Tables**

**Table S1.** List of primers used for PCR of WT and truncated versions of KP34gp57 protein

| **Cloning primers** | **Forward** | **Reverse** |
| --- | --- | --- |
| **WT** | ATGGCACTCACTAAACTAGTAG | ACCAGTGAGTTCAGATGGAG |
| **N-K5** | ATGAAACTAGTAGATGCAGGCG | ACCAGTGAGTTCAGATGGAG |
| **N-D8** | ATGGATGCAGGCGCCTGGCAAG | ACCAGTGAGTTCAGATGGAG |
| **N-A11** | ATGGCCTGGCAAGTAGAAGTAGC | ACCAGTGAGTTCAGATGGAG |
| **N-V14** | ATGGTAGAAGTAGCCCCAGCTGG | ACCAGTGAGTTCAGATGGAG |
| **N-A17** | ATGGCCCCAGCTGGCACAGTAC | ACCAGTGAGTTCAGATGGAG |
| **N-G20** | ATGGGCACAGTACAGGATTCCC | ACCAGTGAGTTCAGATGGAG |
| **N-Q23** | ATGCAGGATTCCCTAGTATTCTTATC | ACCAGTGAGTTCAGATGGAG |
| **N-L26** | ATGCTAGTATTCTTATCCCCACGC | ACCAGTGAGTTCAGATGGAG |
| **N-L29** | ATGTTATCCCCACGCAACTTCGG | ACCAGTGAGTTCAGATGGAG |
| **N-R32** | ATGCGCAACTTCGGAGGAGTAC | ACCAGTGAGTTCAGATGGAG |
| **N-G35** | ATGGGAGGAGTACCAGGCACG | ACCAGTGAGTTCAGATGGAG |
| **N-T40** | ATGACGGGCGTAGATAGTGTAGC | ACCAGTGAGTTCAGATGGAG |
| **N-V45** | ATGGTAGCAGCTATAGAAGCCGC | ACCAGTGAGTTCAGATGGAG |
| **N-A50** | ATGGCCGCCCTAGCGGCAGG | ACCAGTGAGTTCAGATGGAG |
| **N-H105** | ATGCACCCTGTATACGTAGACC | ACCAGTGAGTTCAGATGGAG |
| **N-V167** | ATGGTTAGTGGTGACACGGTG | ACCAGTGAGTTCAGATGGAG |
| **N-M267** | ATGGCGTTTGGTAGTCACGAC | ACCAGTGAGTTCAGATGGAG |
| **C-G581** | ATGGCACTCACTAAACTAGTAG | ACCGGCCCTACCGATAGTGG |
| **C-T522** | ATGGCACTCACTAAACTAGTAG | GGTGCTGCCGCTGATTACGA |
| **C-L469** | ATGGCACTCACTAAACTAGTAG | CAGTACTGCGGTTCTACCCAG |

| KP34gp57  mutants | Mutagenic primer F | Mutagenic primer R | Lengh (aa) |
| --- | --- | --- | --- |
| R145L | GCCTCAGTAGTACCCTGGGGATTATTGGCAG | CTGCCAATAATCCCCAGGGTACTACTGAGGC | 630 |
| D151A | GGATTATTGGCAGCGCTGCTGTGCTGGTTCC | GGAACCAGCACAGCAGCGCTGCCAATAATCC | 630 |
| D179A | TGGACGCACCAATAGCCACTACGGAGACCTT | AAGGTCTCCGTAGTGGCTATTGGTGCGTCCA |  |
| D219A | AGGTTGATACCTGGGCCTACTGGACCGCTGAT | ATCAGCGGTCCAGTAGGCCCAGGTATCAACCT | 630 |
| D219N | GAGGTTGATACCTGGAATTACTGGACCGCTG | CAGCGGTCCAGTAATTCCAGGTATCAACCTC | 630 |
| Y220A | GTTGATACCTGGGATGCCTGGACCGCTGATTC | GAATCAGCGGTCCAGGCATCCCAGGTATCAAC | 630 |
| D224A | ATTACTGGACCGCTGCTTCCGCCACCTTCGA | TCGAAGGTGGCGGAAGCAGCGGTCCAGTAAT | 630 |
| E266A | GTAACAAAGCCTCTGCGATGGCGTTTGGTAG | CTACCAAACGCCATCGCAGAGGCTTTGTTAC | 630 |
| E266Q | AGTAACAAAGCCTCTCAGATGGCGTTTGGTA | TACCAAACGCCATCTGAGAGGCTTTGTTACT | 630 |
| E300A | GTATTTCCTGGGCTGCGTCAGGGCGGCGCTG | CAGCGCCGCCCTGACGCAGCCCAGGAAATAC | 630 |
| E300Q | GGTATTTCCTGGGCTCAGTCAGGGCGGCGCT | AGCGCCGCCCTGACTGAGCCCAGGAAATACC | 630 |
| D382A | CCGCGGTGGTTGTGGACGTATATAAATCCGCT | AGCGGATTTATATACGTCCACAACCACCGCGG | 630 |
| D382N | GCCGCGGTGGTTGTGGACGTATATAAATCCG | CGGATTTATATACGTCCACAACCACCGCGGC | 630 |
| Y384A | GTGGTTGTGGACGTAGCTAAATCCGCTAACGA | TCGTTAGCGGATTTAGCTACGTCCACAACCAC | 630 |
| D389A | ATAAATCCGCTAACGCTTCGGCTATTAATGC | GCATTAATAGCCGAAGCGTTAGCGGATTTAT | 630 |

**Table S2.** List of primers used for PCR and site-directed mutagenesis of KP34gp57

**Table S3.** Proteins similar to KP34gp57 and LKA1gp49 containing a β-barrel insertion domain

| **Name, accession number, PDB id, taxonomy, specificity and activity, reference** | **Proteins with high sequence identity containing a β-barrel insertion domain** |
| --- | --- |
| **KP34gp57** | **29-45% sequence identity with KP34gp57** |
| YP_003347651.1 | Caulobacteraceae bacterium (MBV9995377.1) |
| - | Cupriavidus necator (WP_148271659.1, WP_171517135.1) |
| Klebsiella phage | Cupriavidus necator N-1 (AEI80363.1) |
| *Autographiviridae; Slopekvirinae; Drulisvirus* | Cupriavidus pinatubonensis (WP_220632229.1) |
| K63 capsular serotype | Escherichia coli (HBB2622442.1, HAH0528580.1, HBA5781921.1, HBC0634688.1) |
| This study | Klebsiella phage Menlow (YP_009796383.1) |
|  | Klebsiella phage KP36 (YP_009226011.1) |
|  | Klebsiella pneumoniae (WP_109217531.1, WP_023286700.1, MBC4451710.1, WP_101817041.1, VGL56835.1, WP_117362104.1, MBD7325691.1, WP_194444692.1, WP_223059151.1, PLN07538.1, WP_228963680.1) |
|  | Klebsiella variicola (WP_206551964.1, WP_182000019.1) |
|  | Pseudomonas helleri (WP_153428563.1) |
|  | Serratia phageKpYy 2 45 (YP_010298456.1)  Bradyrhizobium sp. (WP_247372564.1, MCK1707678.1, WP_247380153.1) |
| **LKA1gp49** | **25-38% sequence identity with LKA1gp49** |
| YP_001522890.1 | Acinetobacter phage APK15 (UAW10027.1) |
| 4RU4 | Bacillus phage Mater (YP_009151108.1 ) |
| Pseudomonas phage | Bacillus phage RadRaab (ASU04188.1) |
| *Autographiviridae; Krylovirinae; Stubburvirus* | Bacillus phage StevenHerd11 (AZF88335.1) |
| B-band O-polysaccharide of LPS | Escherichia phage vB_EcoM_APEC (QMP81465.1) |
| the O5-serotype polysaccharide lyase | Escherichia phage YZ1 (YP_009798557.1) |
| Olszak et al. 2017 | Podoviridae sp. (DAX11679.1, DAG73134.1, DAG11756.1) |
|  | Podoviridae sp. Ct9f93 (DAD92677.1) |
|  | Pseudomonas (WP_203308027.1) |
|  | Pseudomonas aeruginosa (WP_235195394.1, HBO0336564.1, MBX6346225.1, WP_221388673.1, HBO1318966.1, WP_180738804.1, WP_010792353.1, MBG5440197.1, WP_058020086.1, WP_134300605.1, WP_126621734.1, HBO1227153.1, WP_134281626.1, WP_023102121.1, WP_058171922.1, WP_073625114.1, WP_034066124.1, WP_057383914.1, MBI8223076.1, WP_108116036.1, HBO8949825.1, WP_208689172.1, MBG4054315.1, WP_052151693.1, HBO3695707.1, WP_058149980.1, WP_116844090.1, WP_134554421.1, WP_188258228.1, WP_134485616.1, HBO5254889.1, MBG6484204.1, WP_237887192.1, WP_124212921.1, WP_208558210.1, WP_096269096.1, MBH3791816.1, MBV5991319.1, HBP5506852.1, WP_023111237.1, WP_134623260.1, WP_125007462.1, WP_034065181.1, HBO1307251.1, WP_058193769.1, HBP5191148.1, WP_096229664.1, WP_198082156.1, WP_058134883.1, WP_225104384.1, MBX5729666.1, WP_087851820.1, WP_198340071.1, HBO5047603.1, HBO2146418.1, MBV5487007.1, WP_241097938.1, WP_058143038.1, WP_079743903.1, MBI8731209.1, WP_121216529.1, WP_121412427.1, HBP5513931.1, EIU5592532.1, WP_171894683.1, WP_121242740.1, MBI8438721.1, WP_124209059.1, OVY90327.1, WP_023093554.1, WP_058172706.1, WP_241360313.1, WP_196654069.1, WP_124131493.1, WP_198089253.1, VFT04322.1, WP_116829901.1, HBN8199355.1, MBX6942390.1, HBP0492519.1, WP_181859006.1, HBP0280394.1, WP_074247552.1, WP_105248003.1, MBG6729090.1, WP_031690859.1, WP_161417231.1, HBP5539535.1) |
|  | Pseudomonas gessardii (NNA92154.1) |
|  | Pseudomonas phage PP9W (UAW06751.1) |
|  | Pseudomonas phage vB_Pae_CF23a (QBI79946.1) |
|  | Pseudomonas rhodesiae (WP_083377850.1) |
|  | Pseudomonas sp. 7-41 (WP_232566324.1) |
|  | Pseudomonas sp. ARP3 (WP_047882142.1) |
|  | Pseudomonas sp. BIS (WP_203327869.1, WP_203306272.1) |
|  | Pseudomonas virus D3 (NP_061523.1) |
|  | Pseudomonas virus PMG1 (YP_005098229.1) |
|  | Siphoviridae sp. (DAL83455.1) |
|  | Variovorax paradoxus (KPU94695.1) |
|  | Vibrio phage 1.167.O._10N.261.51.F2 (AUR91921.1) |
|  | Vibrio phage PS15B.3 (QZI90829.1) |
|  | Vibrio phage PS65A.1 (QZI93320.1 ) |
|  | Vibrio phage PS65A.1 (QZI93320.1 ) |

**Table S4.** The activity of truncated N-A17 and N-Q23 KP34gp57 variants after storage at different temperatures*

| Conditions | | WT MHFC  (nM) | Activity compared to fresh protein (FP_wt_) | N-A17 MHFC  (nM) | Activity compared to fresh protein (FP_N17_) | N-Q23 MHFC  (nM) | Activity compared to fresh protein (FP_N23_) |
| --- | --- | --- | --- | --- | --- | --- | --- |
| 24h | 37 °C | 46.8 | ~ 0.5 FP_wt_ | 1.5 | ~ FP_N17_ | 1.5 | ~ FP_N23_ |
|  | RT | 23.4 | ~ FP_wt_ | 1.5 | ~ FP_N17_ | 1.5 | ~ FP_N23_ |
|  | 4 °C | 23.4 | ~ FP_wt_ | 1.5 | ~ FP_N17_ | 1.5 | ~ FP_N23_ |
|  | -20 °C | 46.8 | ~ 0.5 FP_wt_ | 3.0 | ~ 0.5 FP_N17_ | 3.0 | ~ 0.5 FP_N23_ |
| 1 week | 37 °C | 187.2 | ~ 0.12 FP_wt_ | 3.0 | ~ 0.5 FP_N17_ | 1.5 | ~ FP_N23_ |
|  | RT | 46.8 | ~ 0.5 FP _wt_ | 3.0 | ~ 0.5 FP_N17_ | 1.5 | ~ FP_N23_ |
|  | 4 °C | 46.8 | ~ 0.5 FP_wt_ | 1.5 | ~ FP_N17_ | 1.5 | ~ FP_N23_ |
|  | -20 °C | 374.4 | ~ 0.06 FP_wt_ | 6.0 | ~ 0.25 FP_N17_ | 6.0 | ~ 0.25 FP_N23_ |
| 1 month | 37 °C | 748.9 | ~ 0.03 FP_wt_ | 6.0 | ~ 0.25 FP_N17_ | 3.0 | ~ 0.5 FP_N23_ |
|  | RT | 93.6 | ~ 0.25 FP_wt_ | 3.0 | ~ 0.5 FP_N17_ | 1.5 | ~ FP_N23_ |
|  | 4 °C | 46.8 | ~ 0.5 FP_wt_ | 3.0 | ~ 0.5 FP_N17_ | 3.0 | ~ 0.5 FP_N23_ |
|  | -20 °C | 1497.7 | ~ 0.015 FP_wt_ | 47.9 | ~ 0.03 FP_N17_ | 48.3 | ~ 0.03 FP_N23_ |

*FP stands for the freshly purified protein

KP34gp57Q23

**Supplementary Figures**

**
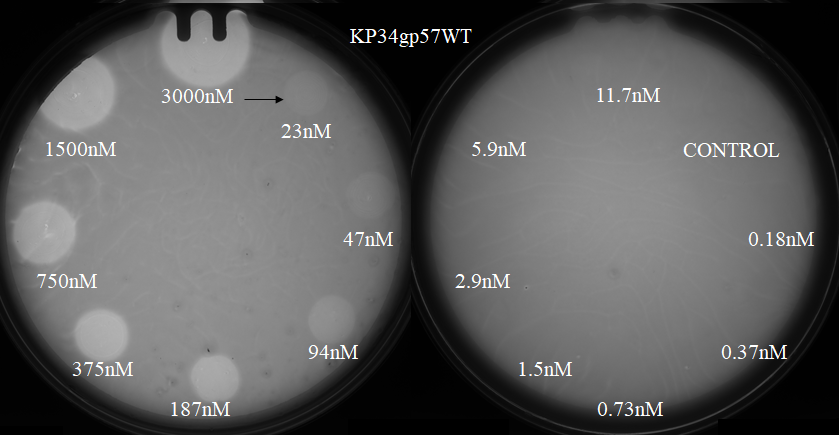

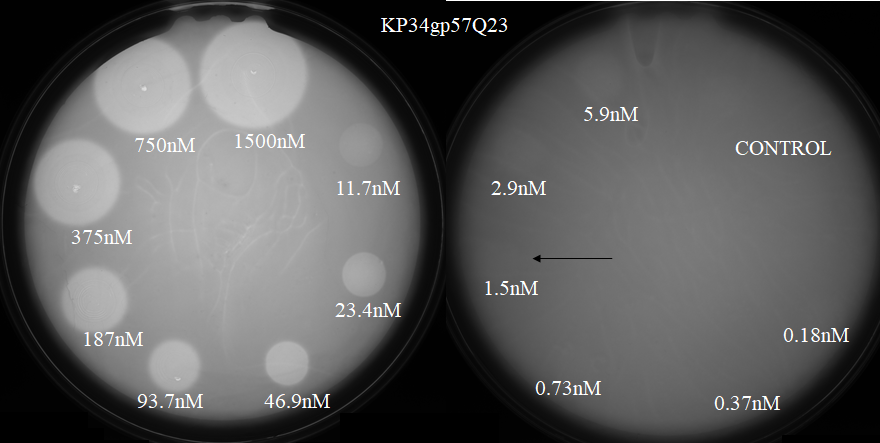

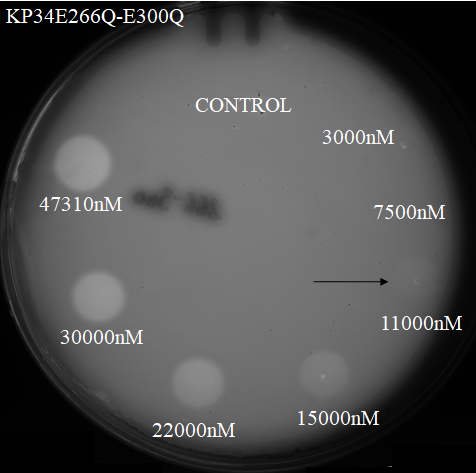
**

C

B

A

**Figure S1.** *K. pneumoniae* K77 strain (K63) lawn on the agar plates with dropped proteins in two-fold serial dilutions: WT depolymerase (A), shorter variant N-Q23 (B), and E266Q-E300Q mutant. The minimal halo-forming unit (MHFC) is indicated by the arrow for each protein.


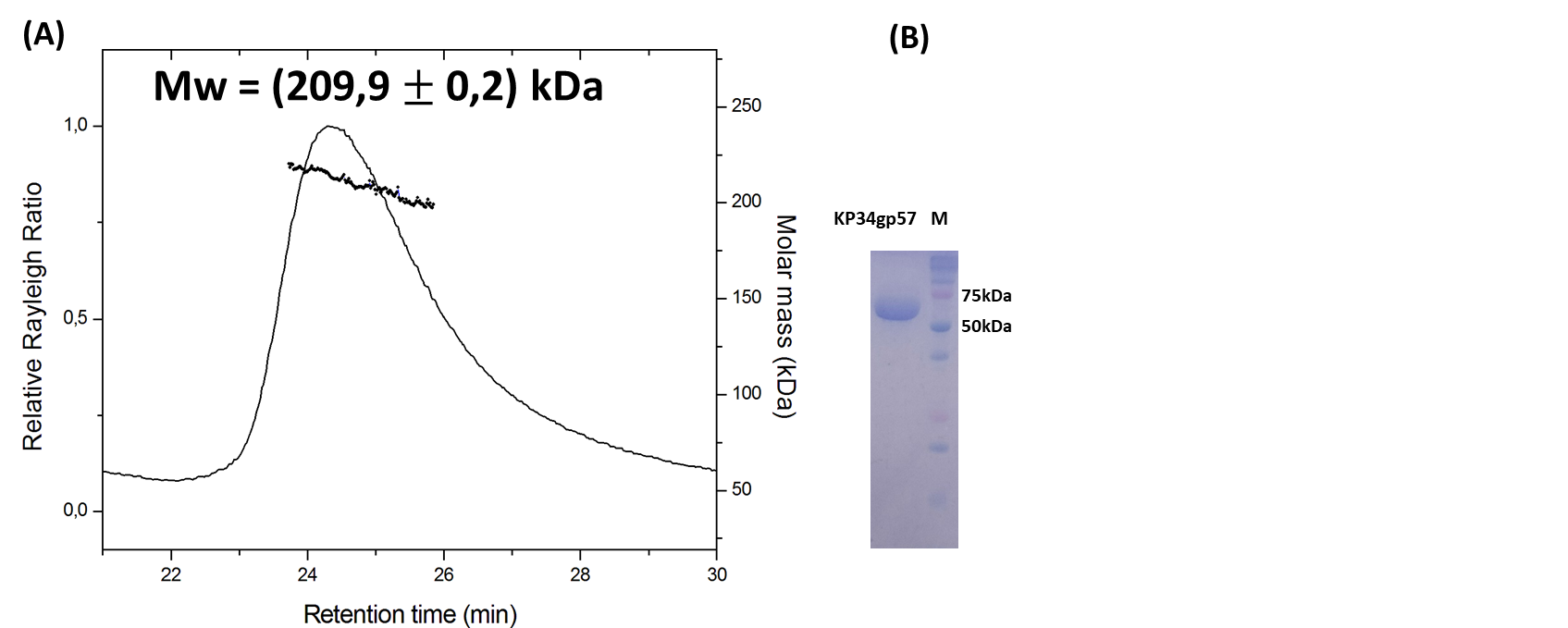


**Figure S2.** (A) Analytical SEC-LS of WT KP34gp57. Relative Rayleigh ratios (left scale) and derived molar masses (right scale) versus elution time. (B) SDS-Page analysis of WT KP34gp57.


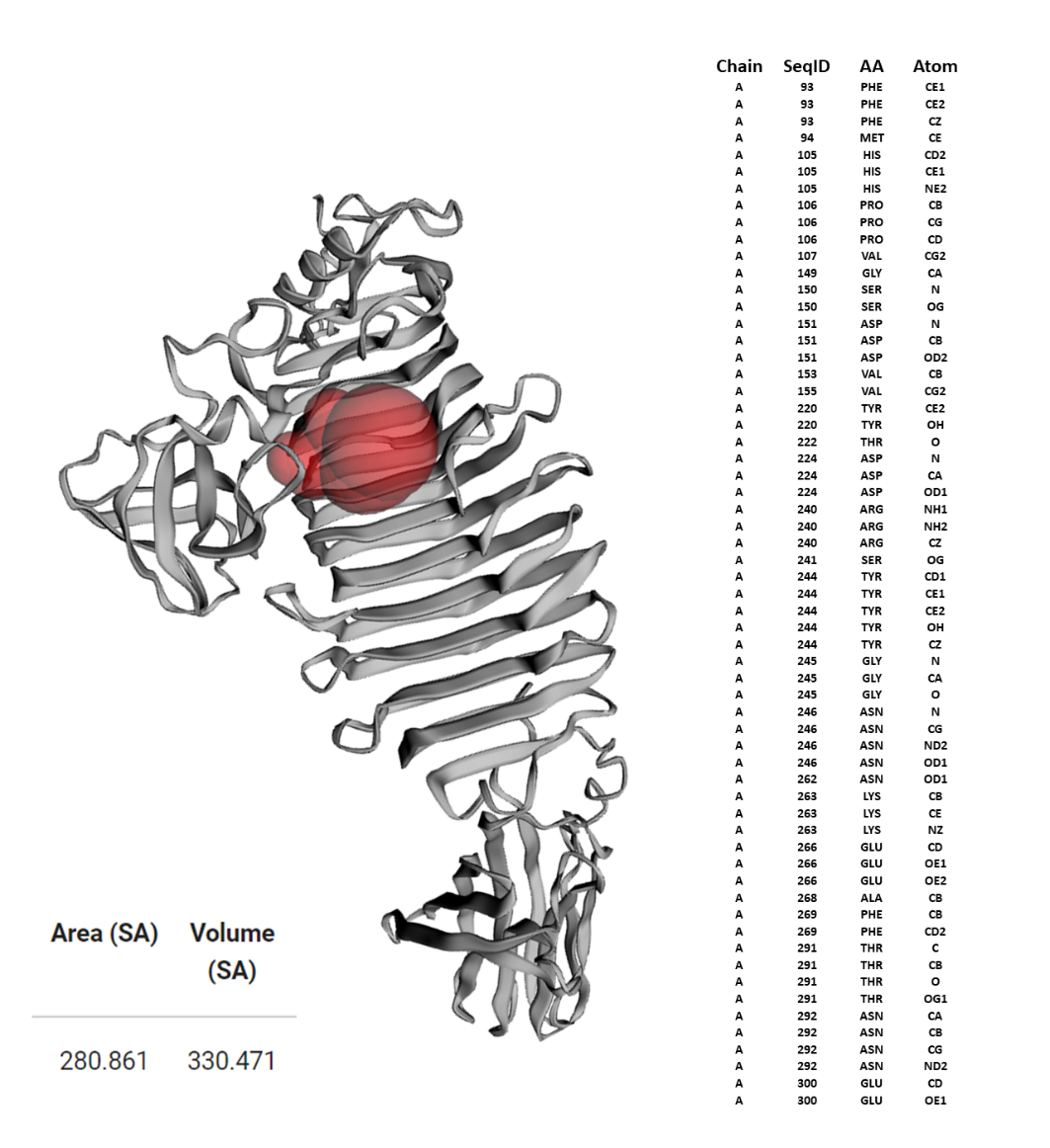


**Figure S3.** Cartoon representation of KP34gp57 chain A and graphic representation of the largest protein cavity, computed using the software CASTp (Computed Atlas of Surface Topography of proteins) (60). Identified residues belonging to this cavity are reported in the table on the right side.

**
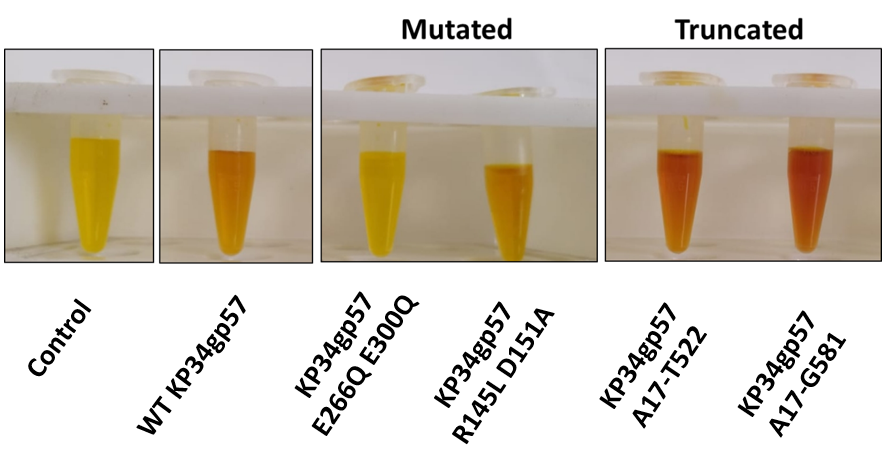
**

**Figure S4.** Samples analysed by the colorimetric method DNS after the enzymatic reaction.


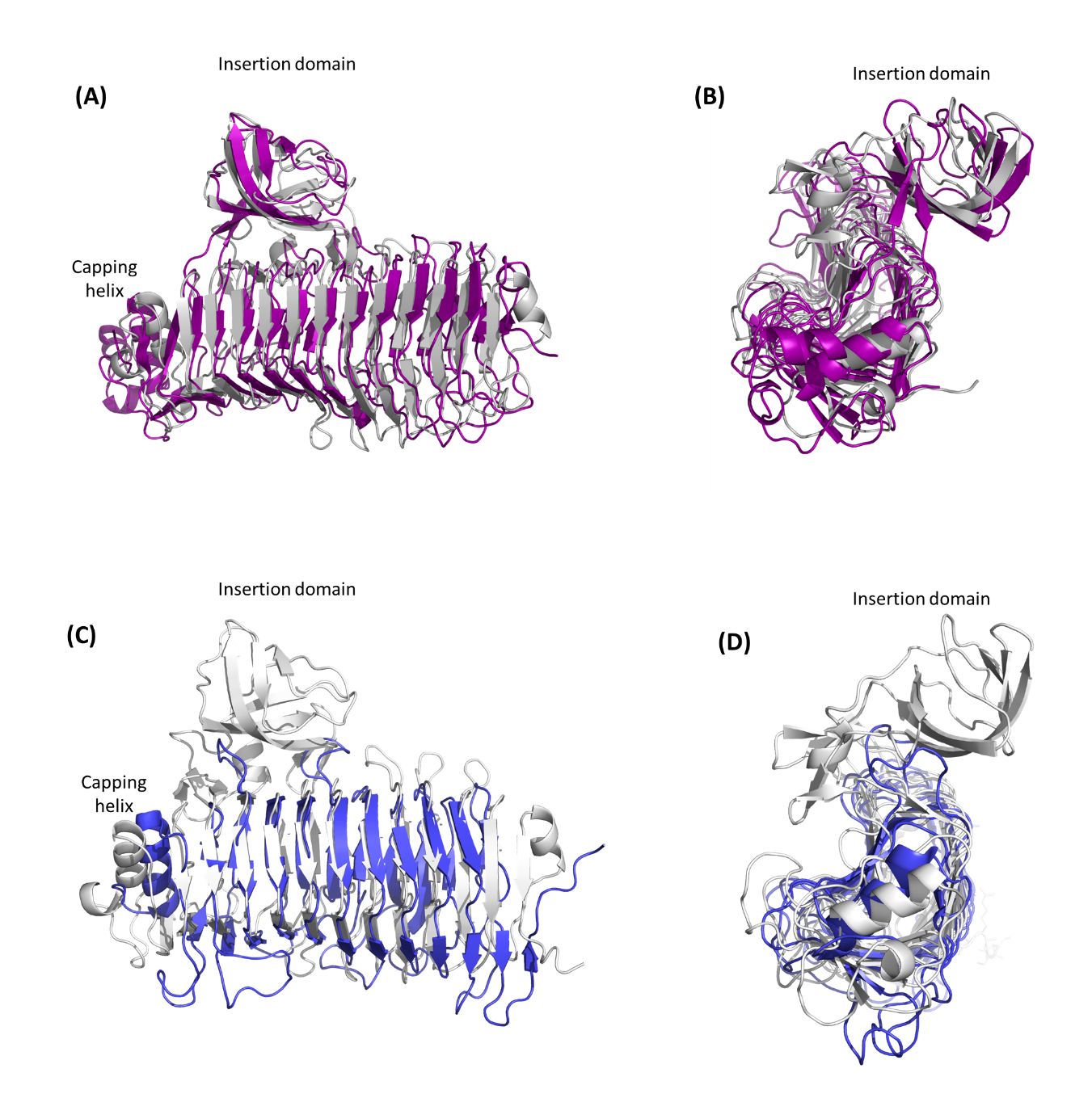


**Figure S5.** Cartoon representation of KP34gp57 (grey) superposed to LKA1gp49 (purple, panels A and B for side and top views, respectively) and KP32gp38 (blue, panels C and D for side and top views, respectively) upon DALI structural superposition.


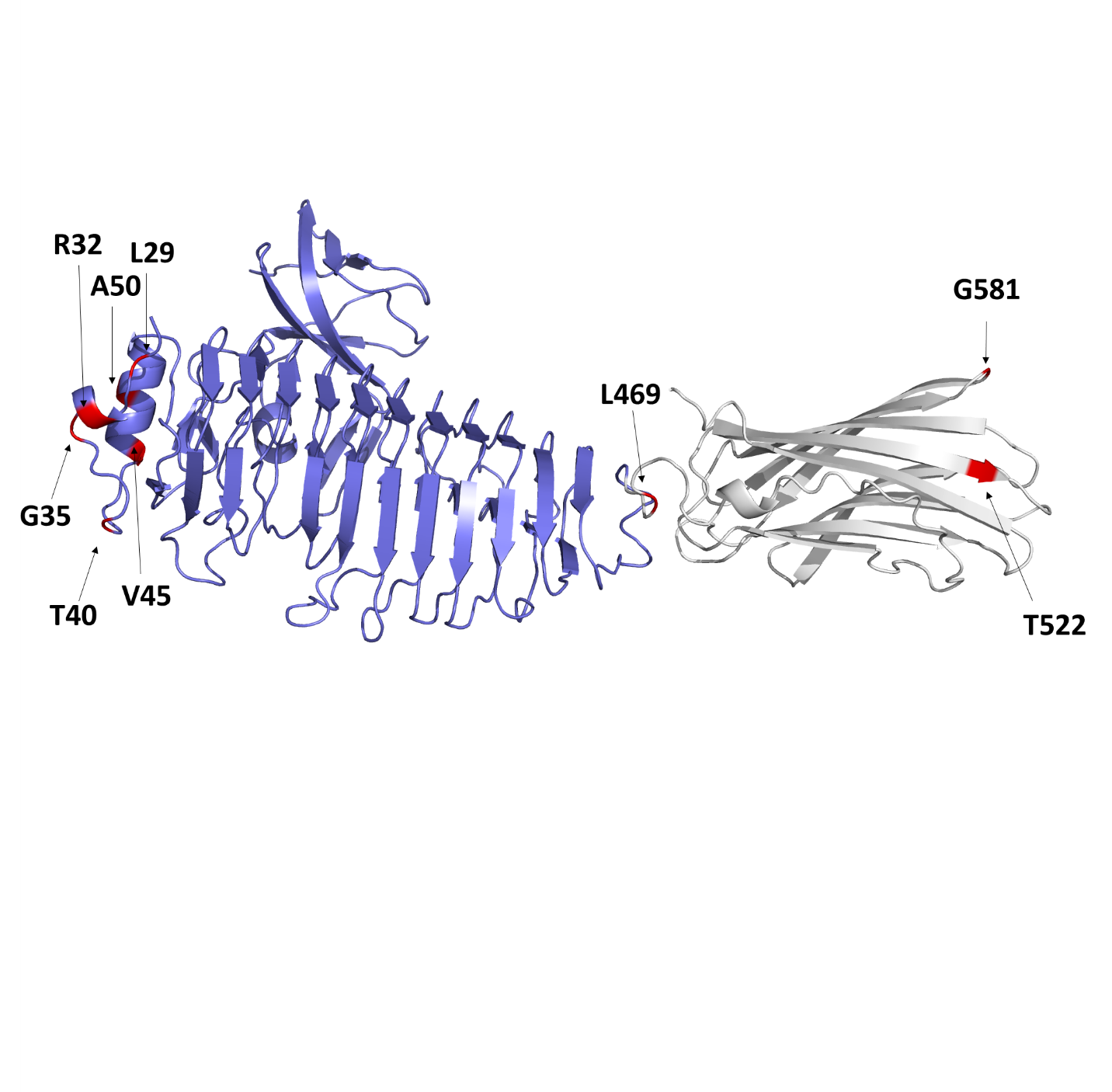


**Figure S6.** Localisation of sites of truncation on the structure of KP34gp57. Truncation sites are coloured red and labelled.


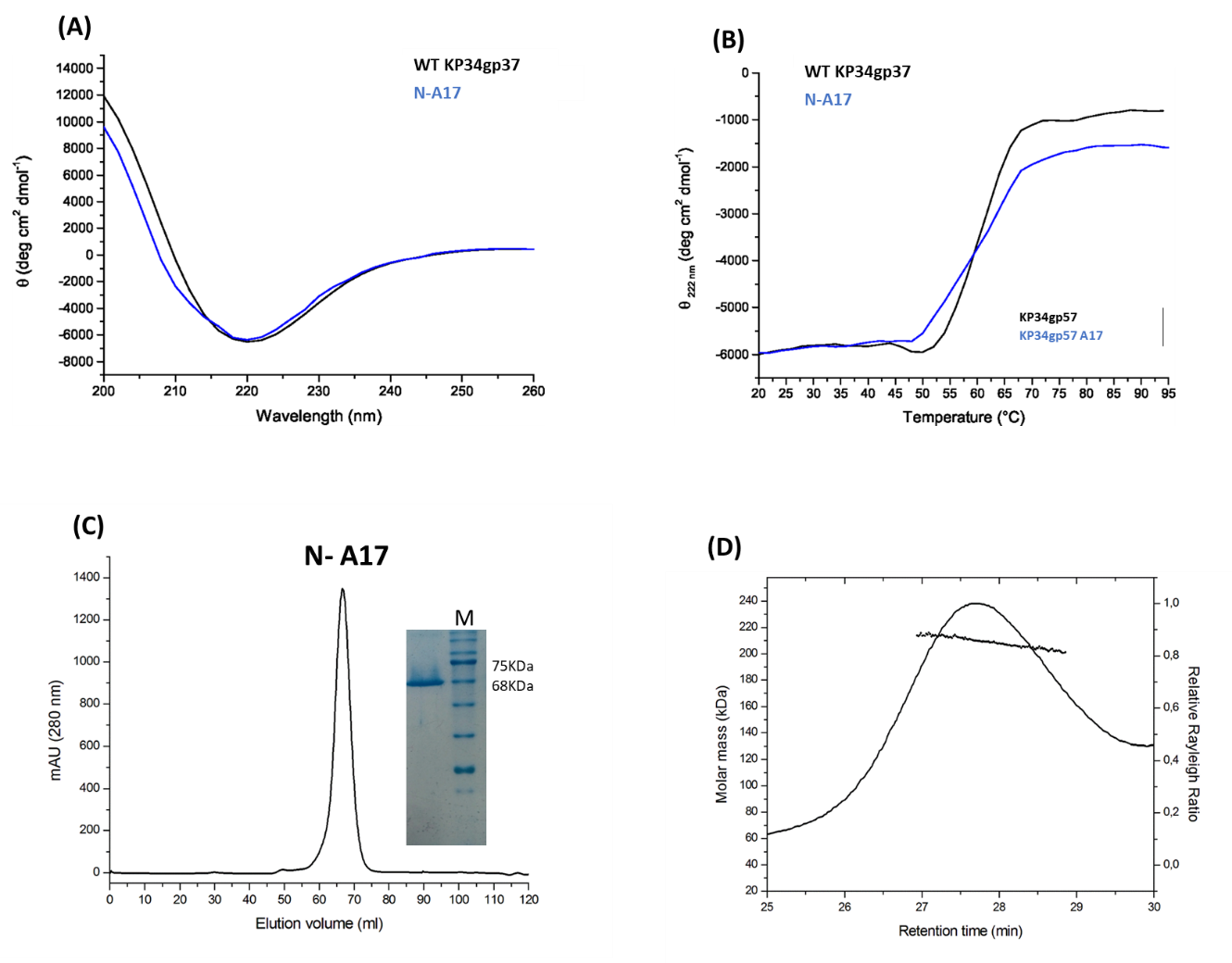


**Figure S7.** Structural characterization in the solution of N-A17 truncated variant of KP34gp57 (N-A17), compared to its wild-type form. (A) Superposition of CD spectra of N-A17 (blue) and wild-type KP34gp57 (black) and (B) of their melting curves, measured at 222 nm. (C) gel chromatography elution profile and SDS-Page showing non-aggregated and pure N-A17; (D) Analytical SEC-LS of N-A17. Relative Rayleigh ratios (left scale) and derived molar masses (right scale) versus elution time;


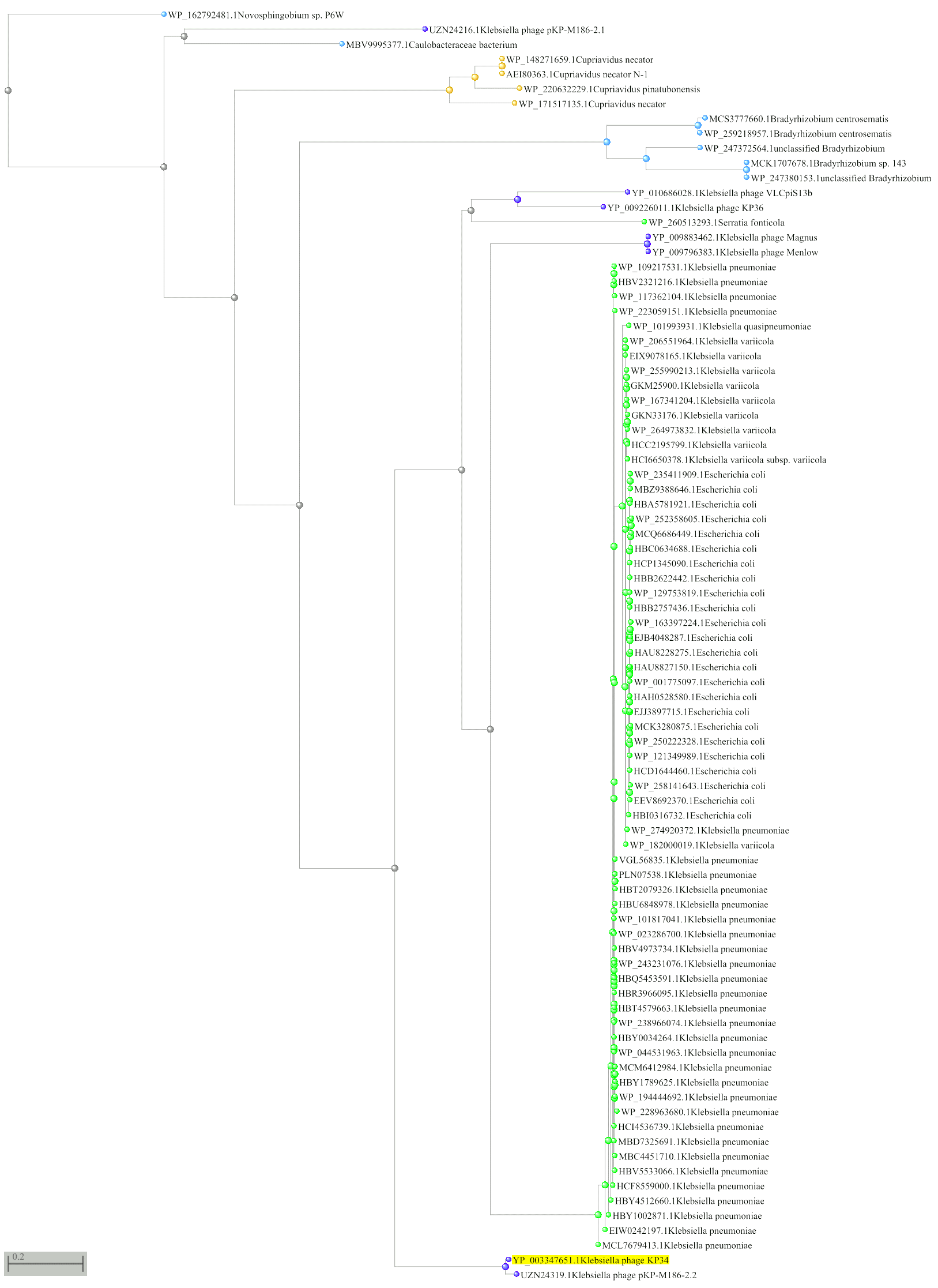


**Figure S8.** The phylogenetic tree of KP34gp57 produced using BLAST pairwise alignments and a function Distance tree of results.


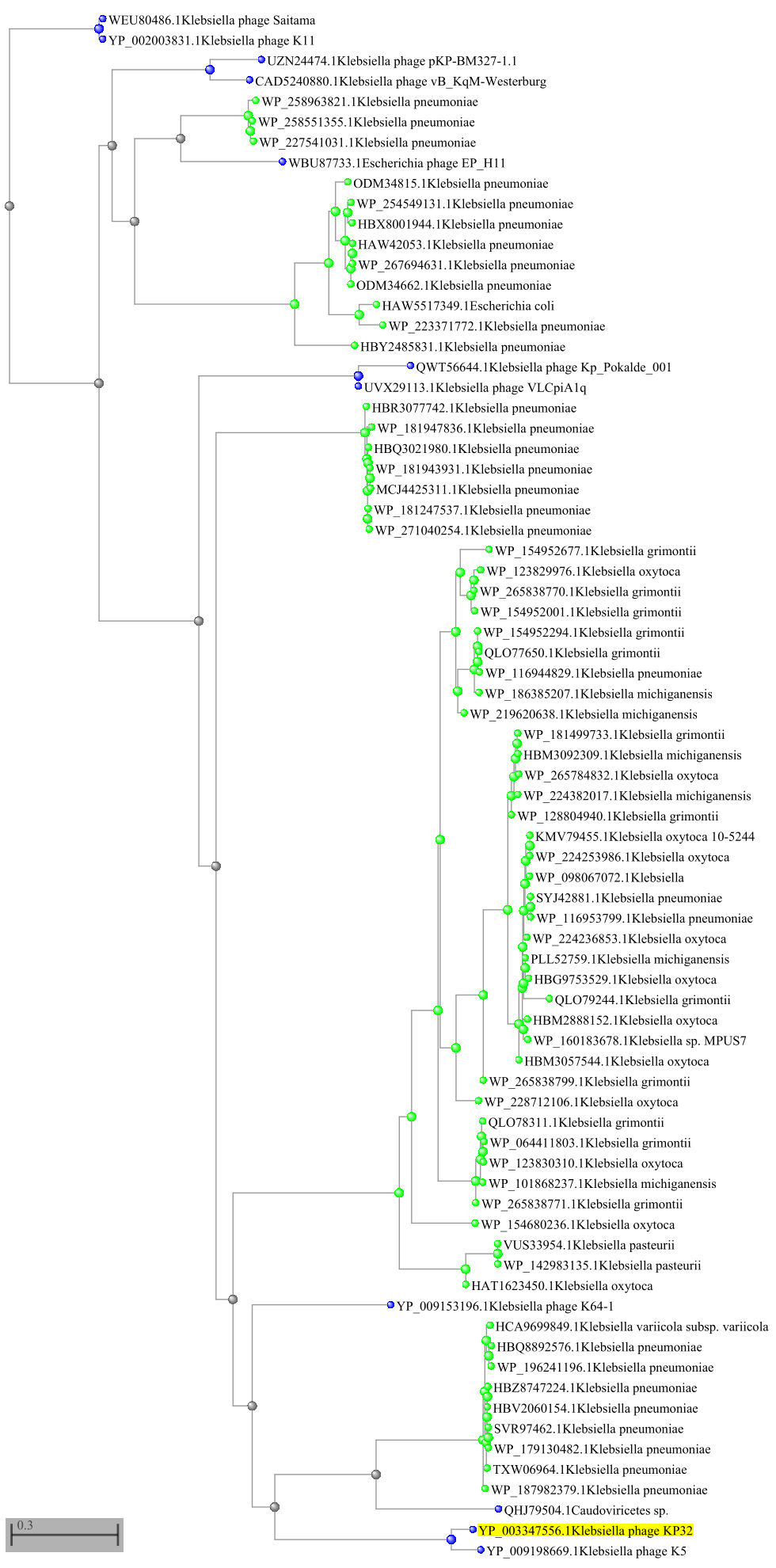


**Figure S9.** The phylogenetic tree of KP32gp38 produced using BLAST pairwise alignments. Viruses and enterobacteria are marked blue and green, respectively.
